# Supplementary material for: Malleable, printable, bondable, and highly conductive MXene/liquid metal plasticine with improved wettability
Source: Nat Commun. 2024 Jul 20;15:6138. doi: 10.1038/s41467-024-50541-4 (PMC11271265; doi:10.1038/s41467-024-50541-4)
Supplement: Supplementary file 1 — Supplementary Information [file 41467_2024_50541_MOESM1_ESM.pdf]

Supplementary Information

Malleable, Printable, Bondable, and Highly Conductive MXene/Liquid Metal Plasticine with Improved Wettability

Haojie Jiang[1], Bin Yuan[1], Hongtao Guo[1], Fei Pan[1], Fanmao Meng[1], Yongpeng Wu[2], Xiao Wang[1], Lingyang Ruan[1], Shuhuai Zheng[1], Yang Yang[1], Zheng Xiu[1], Lixin Li[1], Changsheng Wu[3, 4, 5, 6], Yongqing Gong[1], Menghao Yang[1], and Wei Lu[1],*

[1]Shanghai Key Lab. of D&A for Metal Functional Materials, School of Materials Science & Engineering, Tongji University, Shanghai 201804, China

[2]School of Materials and Chemistry, University of Shanghai for Science and Technology, Shanghai 200093, China

[3]Department of Materials Science and Engineering, National University of Singapore, Singapore, 117575, Singapore

[4]Department of Electrical and Computer Engineering, National University of Singapore, Singapore, 117583, Singapore

[5]Institute for Health Innovation and Technology, National University of Singapore, Singapore, 117599, Singapore

[6]The N.1 Institute for Health, National University of Singapore, Singapore, 117456, Singapore

The PDF file includes:

Supplementary Methods

Supplementary Fig. 1 to 29

Supplementary Table 1 to 3

References

Other Supplementary Material for this manuscript includes the following:

Supplementary Movie 1 to 8

Supplementary Methods

Printing process

The printing of MLM-S plasticine on a PET plate was performed by a Flexible Electronics Printer (MP1100, Shanghai Mifang Electronic Technology Co. Ltd). The extrusion of the plasticine was controlled by an air-powered fluid dispenser and specific parameters are as follows: Nozzle diameter d_1: 840 μm, pressure of 80 KPa, and moving speed of 8 mm s^{-1}; Nozzle diameter d_2: 620 μm, pressure of 90 KPa, and moving speed of 3 mm s^{-1}; Nozzle diameter d_3: 410 μm, pressure of 90 KPa, and moving speed of 2 mm s^{-1}.

Molecular dynamics simulation

The first-principles calculations based on DFT were carried out by using the projector augmented wave (PAW) method as implemented in the Vienna ab initio Simulation Package (VASP)[1]. The correlation energy and exchange energy were calculated by using the Perdew-Burke-Ernzerhof (PBE) functional of the generalized gradient approximation (GGA)[2]. The energy cutoff for plane-wave expansion of the PAWs was 520 eV. The convergence criteria for electronic self-consistent iteration and force were 10-5 eV and 0.02 eV/Å. Vacuum spacing larger than 15 Å was introduced to avoid artificial interaction between the periodic images along the z-direction. In addition, the variation of the interaction relationship at the two-phase interface was simulated using AIMD. For AIMD simulations, a 1×1×1 k-grid was used. The amorphous structure of gallium indium alloy obtained by melting annealing at 800 K-300 K was simulated by AIMD, and then the AIMD was re-performed by combining with the substrate. Considering the actual simulation situation, the efficiency at 300 K was very low, so the simulation temperature was appropriately increased to 500 K under the NVT (Nose-

Hoover thermostat) ensemble. We used a 2 fs step in the AIMD simulations. Visualization analyses were carried out using VESTA and OVITO[3,4].

Preparation of EMI shielding testing samples and EMI shielding testing

Experimental weighing paper (Titan; 20 μm thick) was coated evenly with MLM by a PTFE blade. The film assembly was cut to a 22.86 mm × 10.16 mm rectangle. EMI shielding performance in the frequency range of 5.88-8.17 GHz, 8.2-12.4 GHz, and 12-18 GHz were measured by waveguide method using a 2-port network analyzer (VNA, 3672B-S, Ceyear, China). The scattering parameters of the reflection coefficient data S_{11} and the transmission data S_{21} were measured to calculate the power coefficients of reflection (R), transmission (T), and absorption (A).

$$R = |S_{11}|^2 = |S_{22}|^2 \quad (1)$$

$$T = |S_{12}|^2 = |S_{21}|^2 \quad (2)$$

$$A = 1 - (T + R) \quad (3)$$

The calculation of total EMI SE (SE_T), reflection efficiency (SE_R), absorption efficiency (SE_A), and multiple reflection efficiency (SE_M) is as follows:

$$SE_T = 10\log\frac{1}{T} \quad (4)$$

$$SE_R = 10\log\left(\frac{1}{1-R}\right) \quad (5)$$

$$SE_A = 10\log\left(\frac{1-R}{T}\right) \quad (6)$$

$$SE_T = SE_R + SE_A + SE_M \quad (7)$$

where SE_M could be ignored when SE_T > 10 dB[5]. To further compare the shielding performance of the materials, the EMI SE is normalized to remove the effects of thickness and density. Taking into account the contributions of density (ρ, g cm^{-3}) and

thickness (t, cm), SSE (dB cm^3 g^{-1}) and $SSEt$ (dB cm^2 g^{-1}) are obtained using the equations:

$$SSE = \frac{SE_T}{\rho} \tag{8}$$

$$SSE_t = \frac{SSE}{t} \tag{9}$$

Characterizations

The phase and crystal structure of samples were tested by X-ray diffraction (XRD, DX-2700) using Cu-Ka radiation (λ = 1.54 Å). The morphology of coatings was characterized by scanning electron microscopy (SEM, secondary electron imaging mode, S-4700, Hitachi, Japan), and the component distribution of the coating was characterized by SEM (Backscattered electron imaging mode, TM4000 plus, Hitachi, Japan). MXene nanosheets were characterized by transmission electron microscopy (TEM, JEM-2100F, 200 kV, Japan). X-ray photoelectron spectroscopy (XPS) was performed on a Thermo Scientific K-Alpha spectrometer. Electrical conductivity was detected by utilizing a standard 4-Point Probe Resistivity Measurement System (RTS-9, Guangzhou Four Probe Technology Co., LTD). Surface temperature and infrared properties were taken by a FLIR camera (HIKMICRO-TPK20). Stretching of the samples was conducted on a universal testing machine (Instron 5565, 5KN) with a 150 mm min-1 loading rate at ambient temperature. The electrical sensing properties of the samples were measured through the change in resistance of the sensor during external strain using a Tektronix DMM4050 digital precision multimeter. The contact angle measuring instrument (JC2000D1, Shanghai Zhongchen Digital Technology Equipment Co.) was used to measure the change in contact angle during mixing LM with MXene. The morphology and phase images were characterized by an atomic force microscope (AFM, Dimension ICON, Bruker, Germany) in tapping mode. The

rheological properties of samples were tested at room temperature by a rotating parallel-plate rheometer (HAAKE MARS 60, Thermo Scientific, USA) equipped with a 20 mm parallel plate. Fourier transform infrared (FT-IR) spectra were measured with an FT-IR spectrometer (INVENIO, Bruker, Germany) by the attenuated total reflection mode.

X-ray microscopy characterization

X-ray microscopy (XRM) was performed with ZEISS Xradia 620 Versa in Carl ZEISS (Shanghai) Co. Ltd. The sample was mounted on the holder with an aluminum tube as the adapter and rotated horizontally by 360 angle degrees, pausing at discrete angles to collect 2D projection images, which were then combined to produce a 3D reconstructed volume dataset. Scanning energy is 140 kV/ 21 W, scanning resolution is 1.82 μm/ voxel size, exposure time is 1 s, and projection number is 2001. Dragonfly software is hired for segmentation. By segmentation, the thickness and distribution of different phases can be expressed in 3D volume.

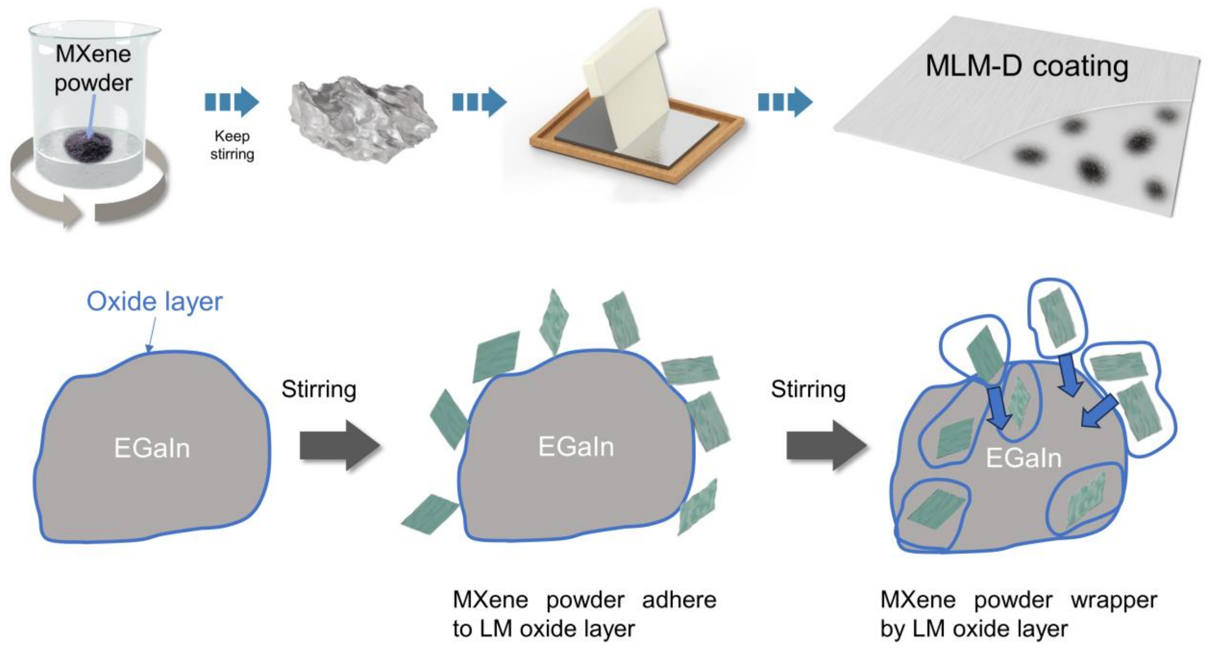

Supplementary Fig. 1 | Fabrication of the MXene/liquid metal composite prepared by dry powder mixing (MLM-D). The illustration of the preparation process and formation mechanism of MLM-D. The above images are all generated by modeling software.

During mechanical stirring, MXene powder is gradually dragged into the LM with the Ga oxide, and the fresh surface of the LM that leaks out is then exposed to air and instantly forms an oxide layer. The continual incorporation of fillers encapsulated with gallium oxide into the interior increases the viscosity of the bulk[6,7].

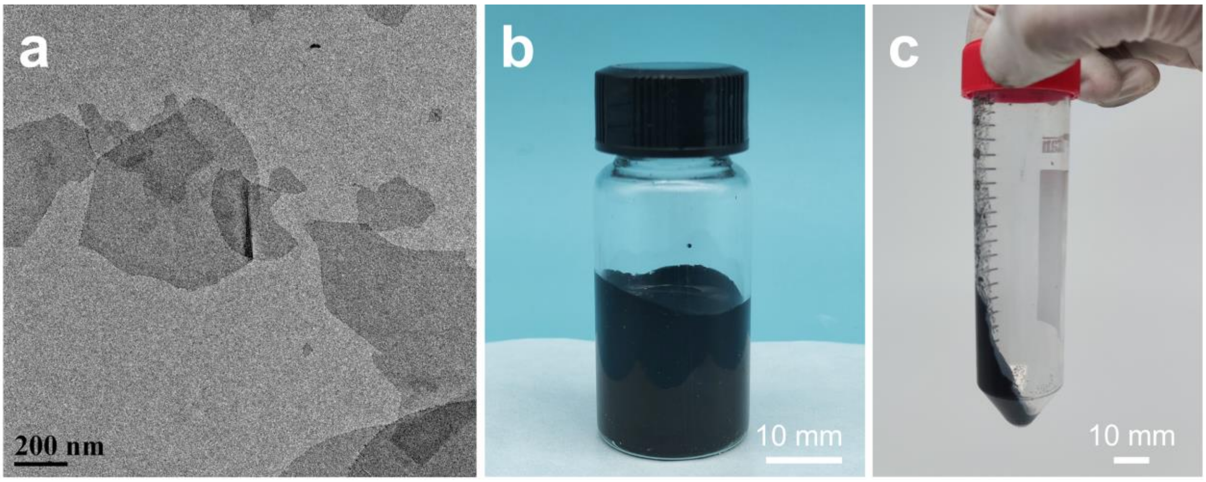

Supplementary Fig. 2 | Microscopic characterization of MXene. (a) TEM images of $Ti_3C_2T_x$ nanosheets. **(b)** $Ti_3C_2T_x$ MXene aqueous dispersion. **(c)** $Ti_3C_2T_x$ MXene paste.

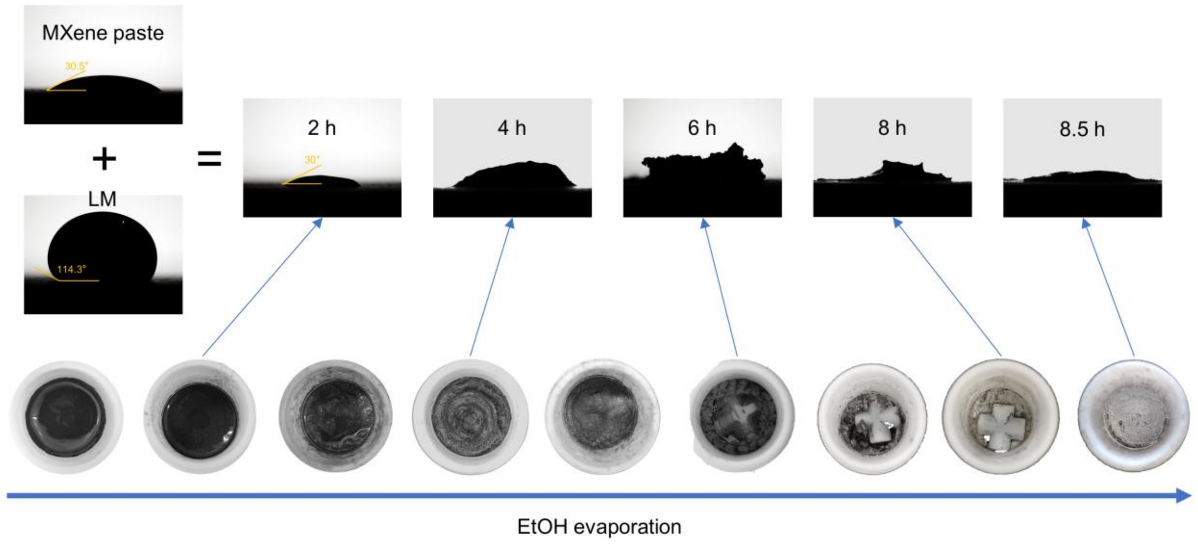

Supplementary Fig. 3 | The contact angle test and the preparation process during solvent-assisted dispersion (SAD). The contact angle and optical photographs of each stage during SAD.

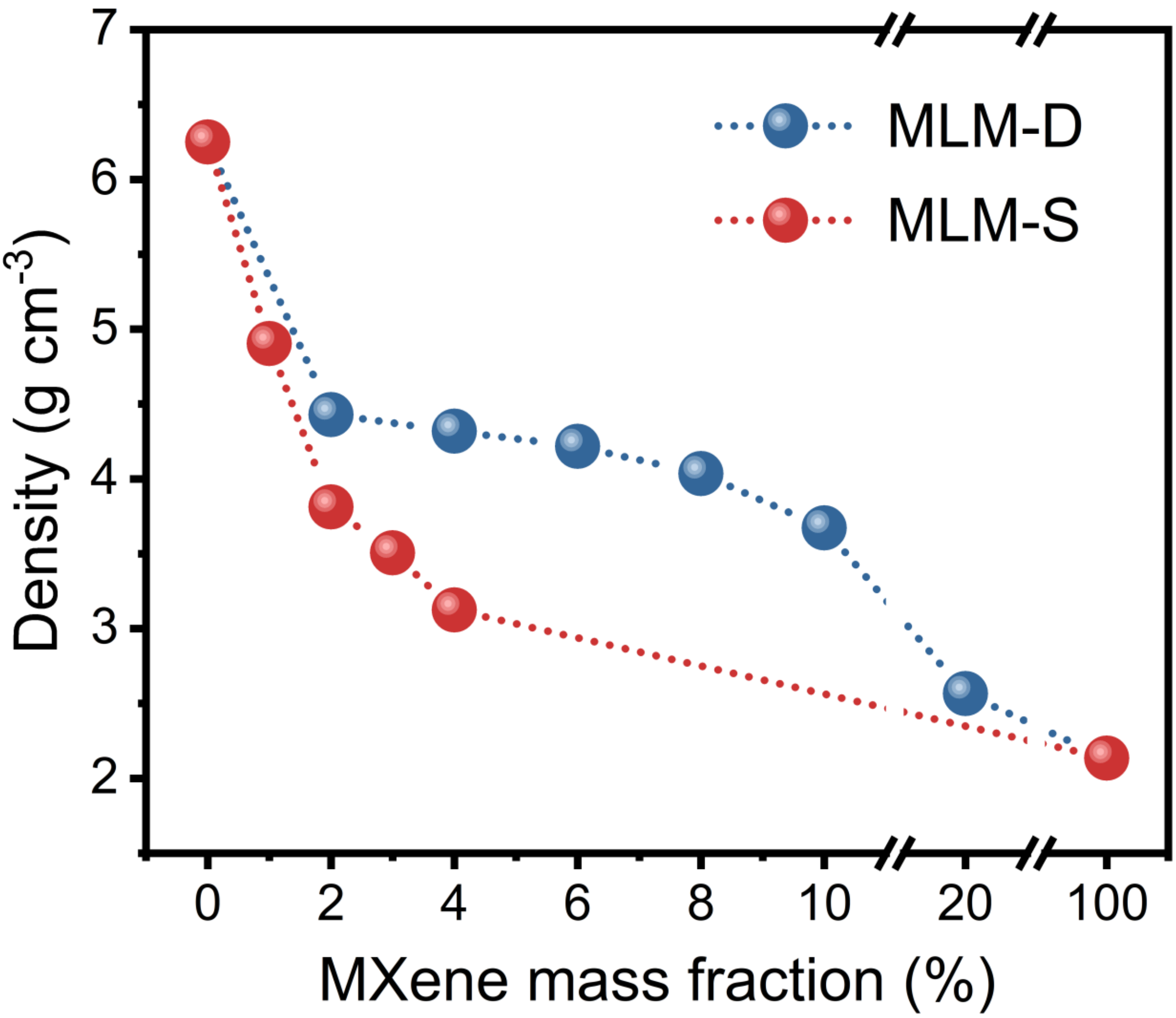

Supplementary Fig. 4 | Densities of samples. Density of MLM as a function of the MXene mass fraction.

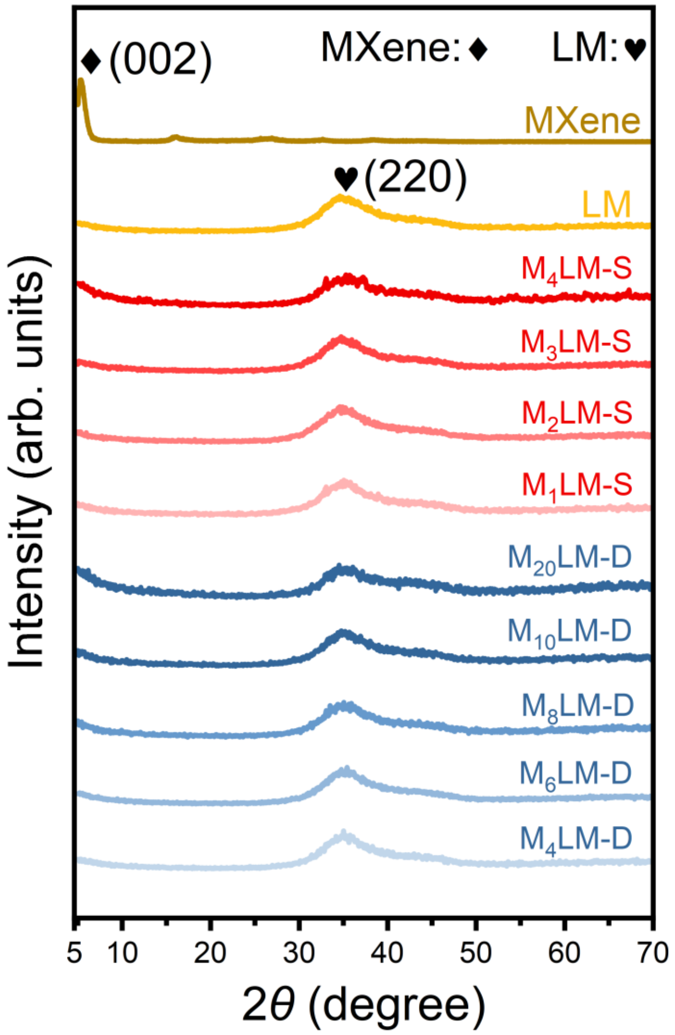

Supplementary Fig. 5 | X-Ray Diffraction (XRD) characterization of samples. XRD patterns curves of raw materials and different ratios of MLM.

Pure MXene exhibited an intense peak at 5.4°, corresponding to the MXene basal plane (002) reflection. LM showed a weak and broad peak in the 30-40° range, corresponding to Ga (220)[8]. According to the XRD spectrum of all MLM, Since most of MXene is covered by metal and metal oxide layers, the characteristic peak signal of MXene (002) in the composite sample is weak. However, the corresponding characteristic peak signals can be captured in samples with high MXene content (M_4LM-S and M_{20}LM-D).

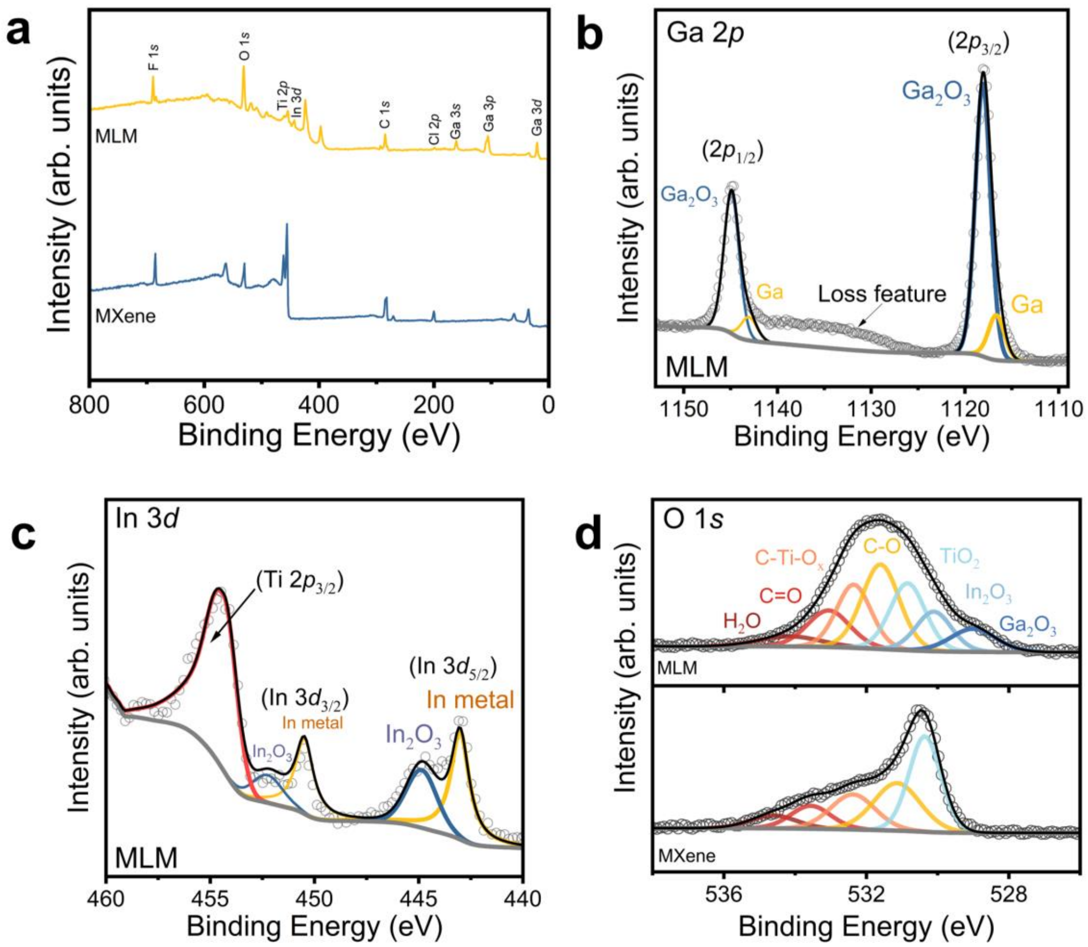

Supplementary Fig. 6 | X-ray photoelectron spectroscopy (XPS) characterization of samples. (a) XPS wide-scan spectra of MXene and MLM. The XPS spectra of **(b)** Ga 2*p* and **(c)** In 3*d* of the MLM composite. **(d)** Comparison of O 1*s* high-resolution XPS spectra of $Ti_3C_2T_x$ and the MLM composite.

The Ga 2*p* and In 3*d* spectra were used to characterize the EGaIn surface oxidation[9]. Metal oxide layers formed on the MLM surface, as indicated by the native Ga_2O_3 and In_2O_3 peaks. In the O 1*s* spectrum, MLM produces more metal oxide subpeaks than the original MXene.

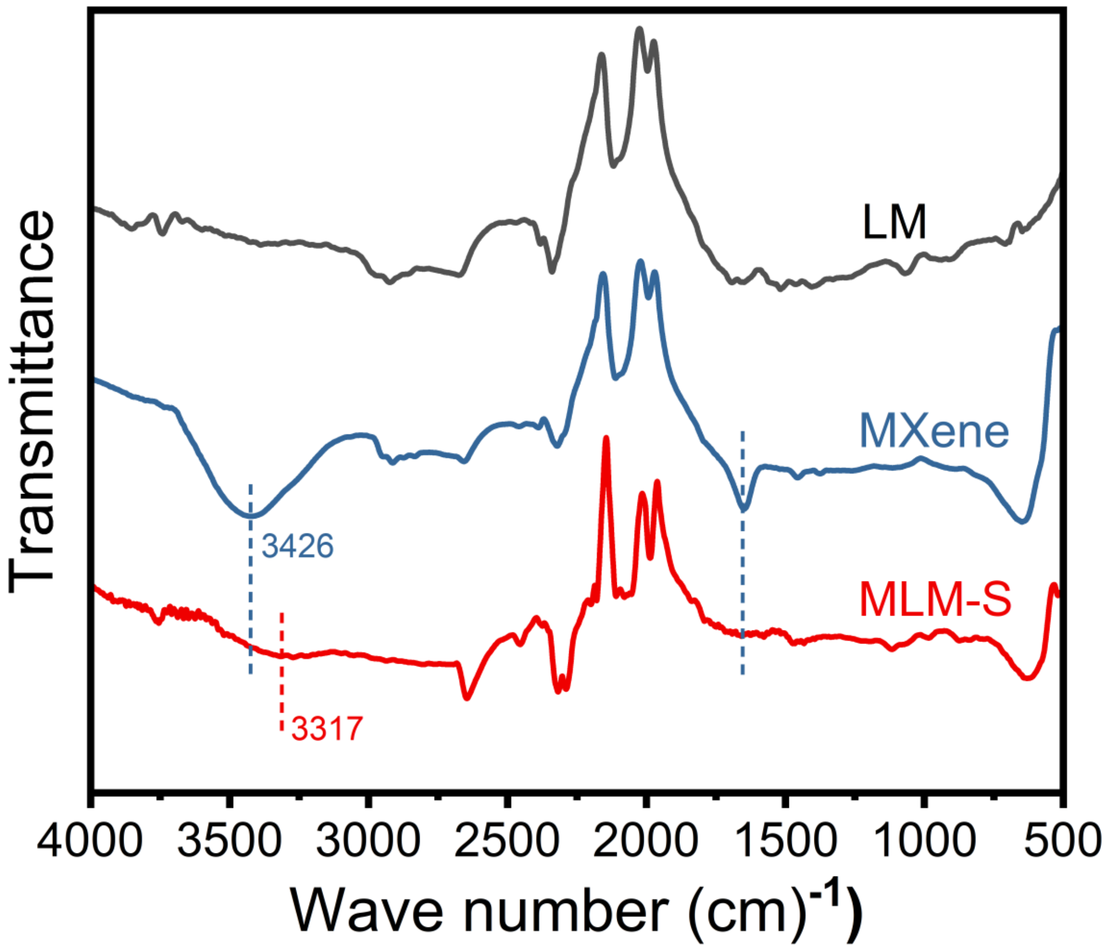

Supplementary Fig. 7 | Fourier Transform Infrared Spectroscopy (FTIR) characterization of samples. FTIR spectra of LM, MXene, and MLM.

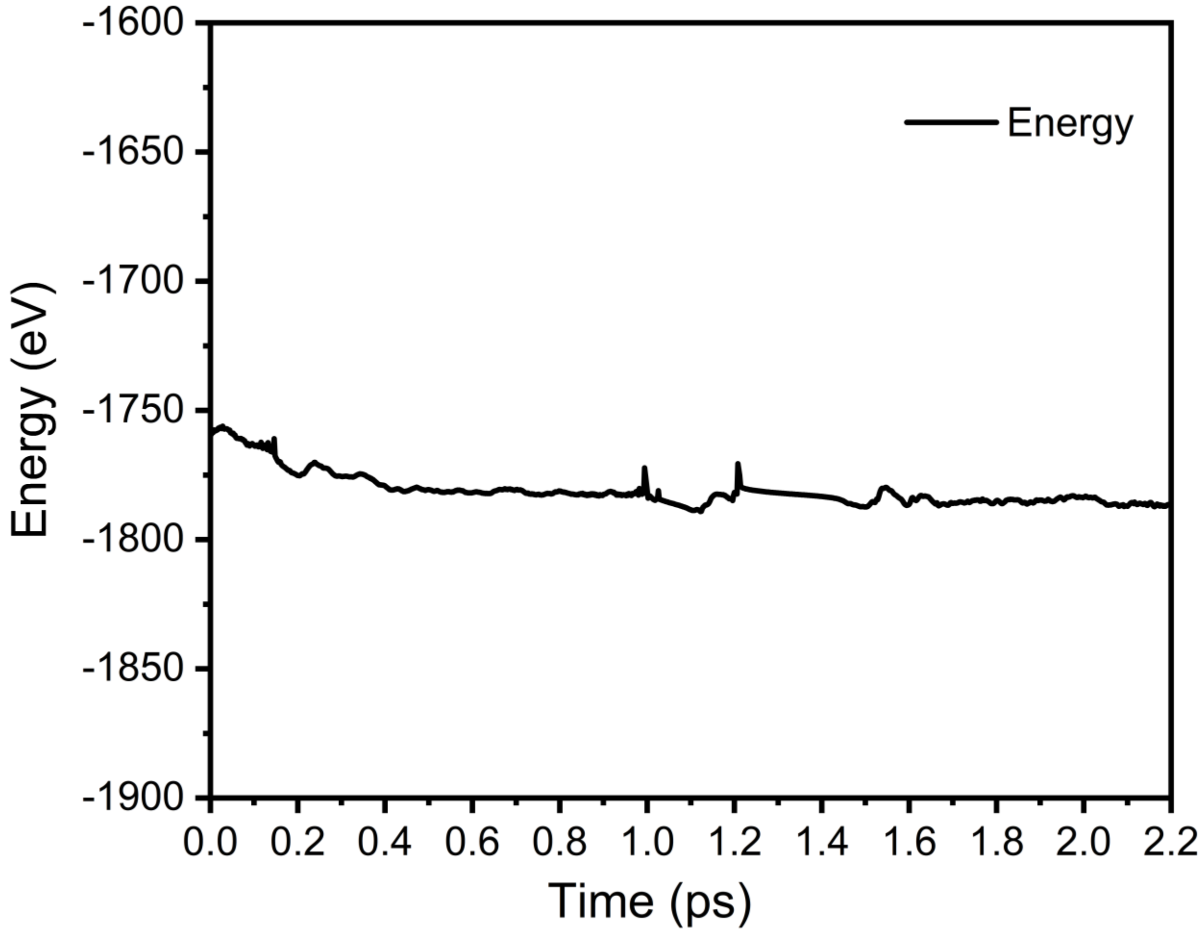

Supplementary Fig. 8 | The system energy change process of Ab initio molecular dynamics (AIMD) process. The system energy variation with time in the AIMD process.

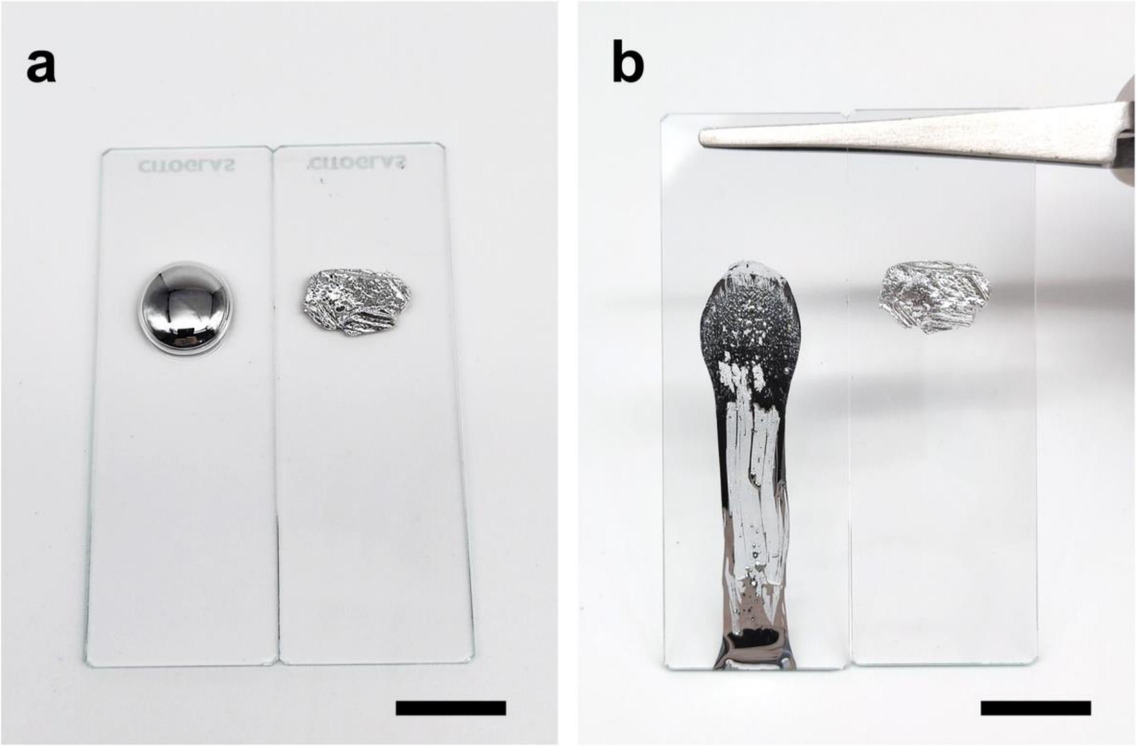

Supplementary Fig. 9 | Comparison between liquid metal (LM) and the MXene/LM composite (MLM). Pure LM and MLM-S on **(a)** flat glass plates and **(b)** tilted glass plates. The scale bar is 10 mm.

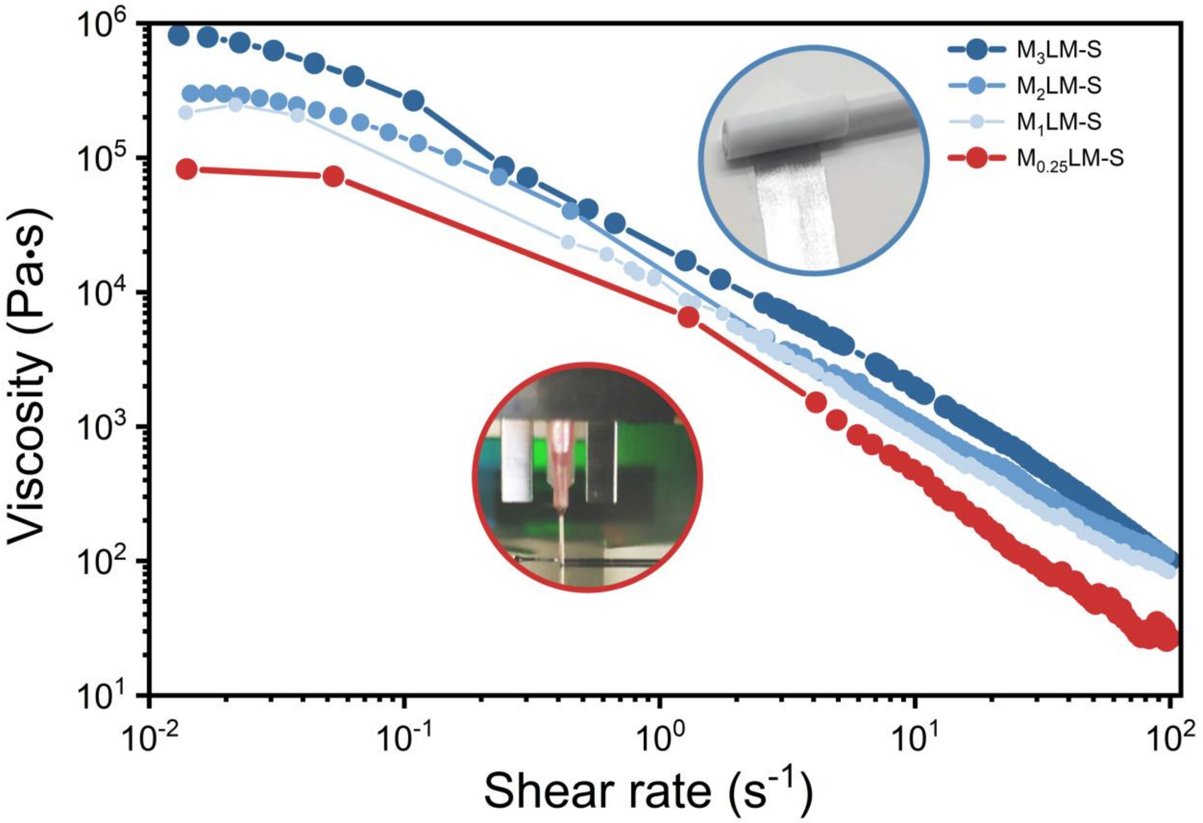

Supplementary Fig. 10 | Rheological test of the MXene/liquid metal composite by solvent-assisted dispersion (MLM-S). Viscosity versus shear rate plots for the MLM-S with different MXene content. Inserts: High-viscosity MLM-S for coating (circled in blue) and low-viscosity MLM-S for printing (circled in red).

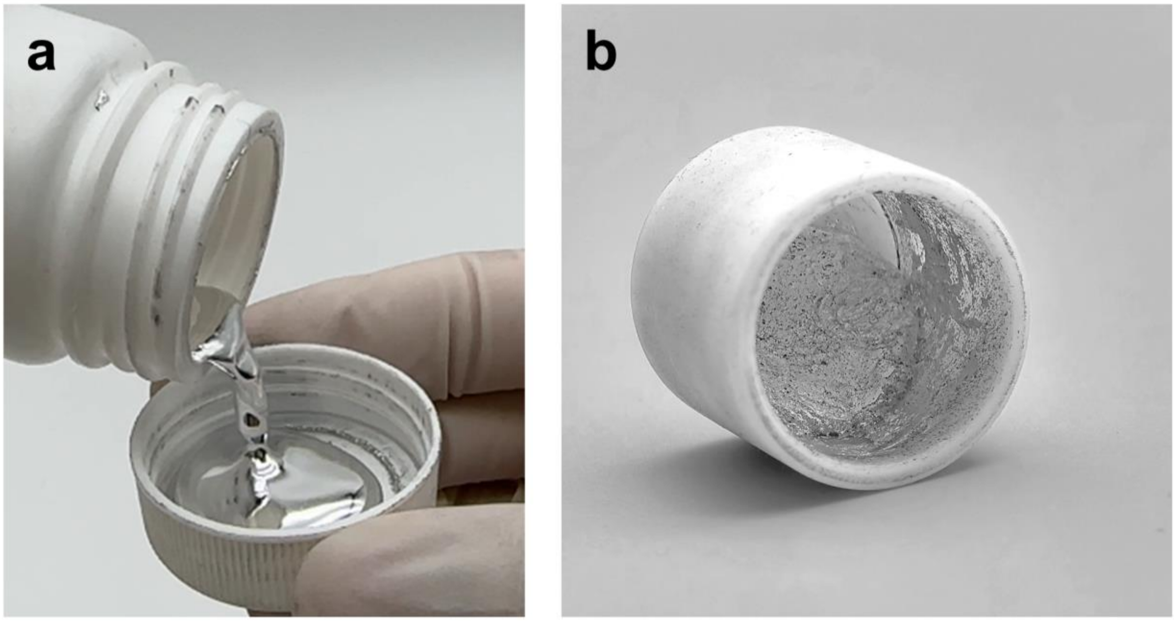

Supplementary Fig. 11 | Comparison of liquidity between liquid metals (LM) and the MXene/liquid metal composite (MLM). (a) Streaming Dynamic LM. **(b)** High viscosity MLM-S.

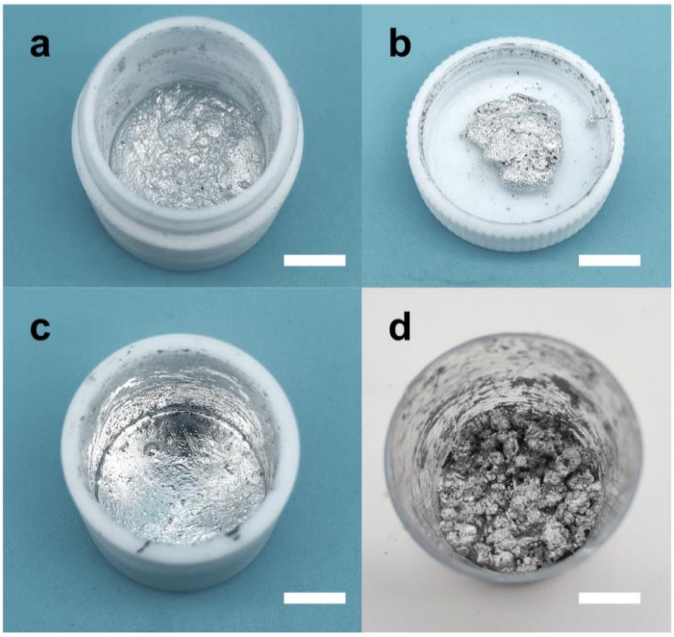

Supplementary Fig. 12 | Macroscopic morphologies of the MXene/liquid metal composites (MLM) with different MXene content. Photos showing LM incorporated with different MXene loading masses. **(a)** M_4LM-D, **(b)** M_{20}LM-D, **(c)** M_2LM-S, **(d)** M_4LM-S. Scale bar, 5 mm.

The maximum weight of infiltrated MXene with SAD is 4.0 wt%, and when it exceeds 4.0 wt%, the viscosity of MLM-S is too high for coating. Correspondingly, the maximum amount of MXene powder infiltration with DPM is 10 wt%, and excessive infiltration can lead to sloughing and cracking.

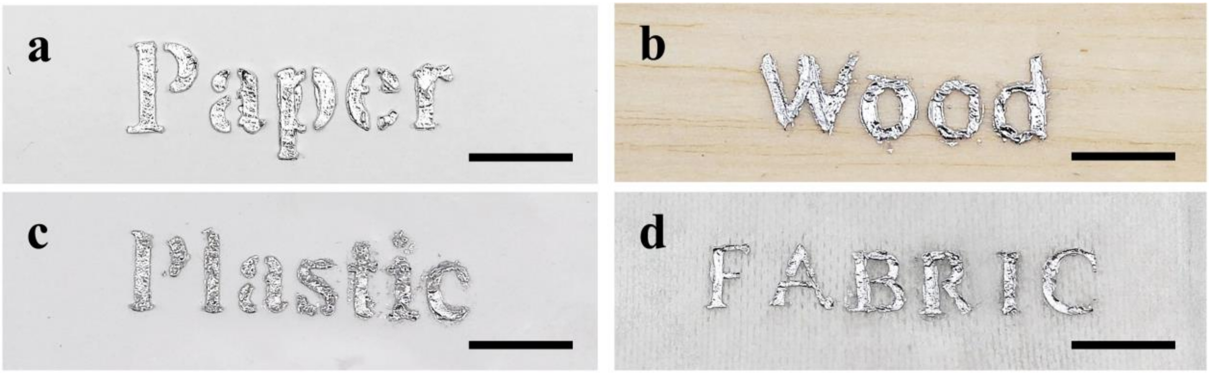

Supplementary Fig. 13 | The wettability of the MXene/liquid metal composite prepared by solvent-assisted dispersion (MLM-S) for various materials. Apply MLM-S to the **(a)** paper, **(b)** wood, **(c)** plastic, and **(d)** fabric. The scale bar is 1 cm.

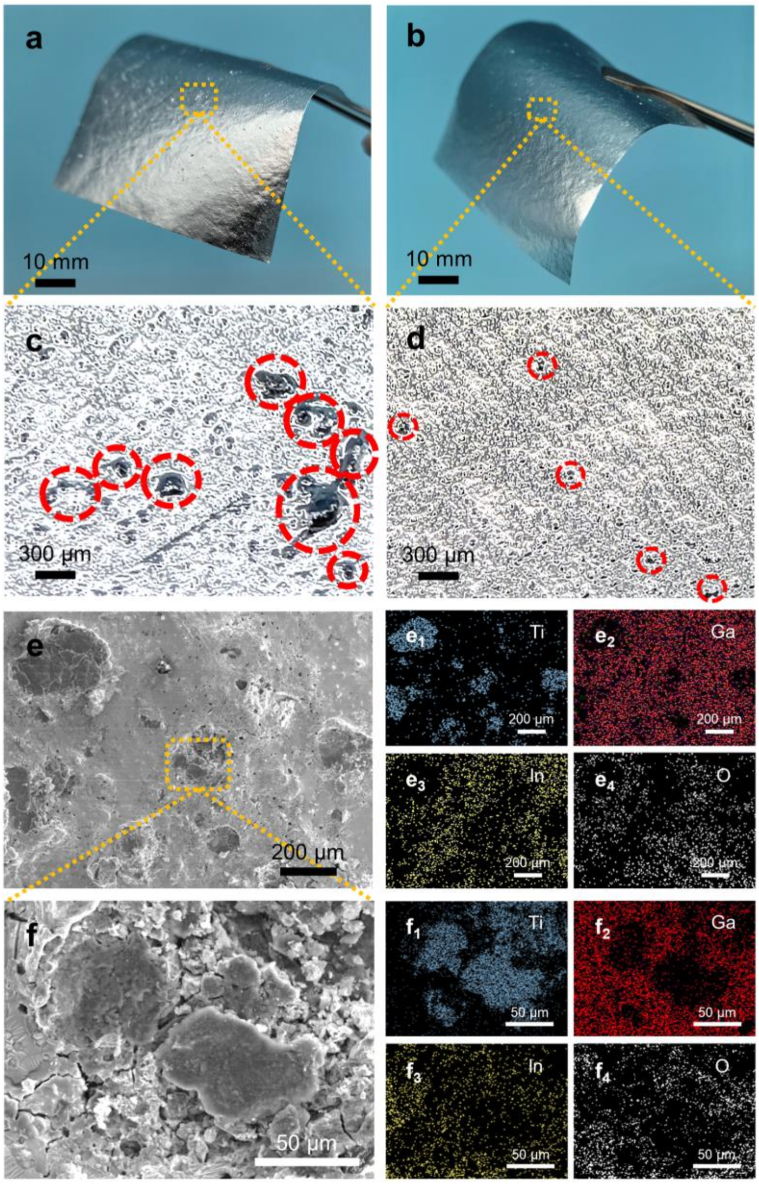

Supplementary Fig. 14 | Microscopic characterization of the MXene/liquid metal composites prepared by dry powder mixing (MLM-D) and solvent-assisted dispersion (MLM-S). (a) The optical photograph of a weighing paper coated with MLM-D. **(b)** The optical photograph of a weighing paper coated with MLM-S. **(c)** Enlarged optical image of the yellow box region in **(a)**. **(d)** Enlarged optical image of the yellow box region in **(b)**. **(e)** SEM image of MLM-D coating. **(e_1-e_4)** EDS mapping of the region of **(e)**. **(f)** Enlarged SEM image of the yellow box region in **(e)**. **(f_1~f_4)** EDS mapping of the region of **(f)**.

Optical photographs of the macroscopic coatings of MLM-D (Supplementary Fig. 14a) and MLM-S (Supplementary Fig. 14b) coatings reveal their dense and uniform,

but the small bumps on the surface of MLM-D are more than those on MLM-S. Under higher magnification in Supplementary Fig. 14c, the bright areas of the surface of MLM-D are dense LM, with large black dots due to the high optical absorption of bare MXene agglomerate (circumscribed in red). In contrast, the surface of MLM-S appears flat and smooth with less diminutive black MXene agglomerates (Supplementary Fig. 14d).

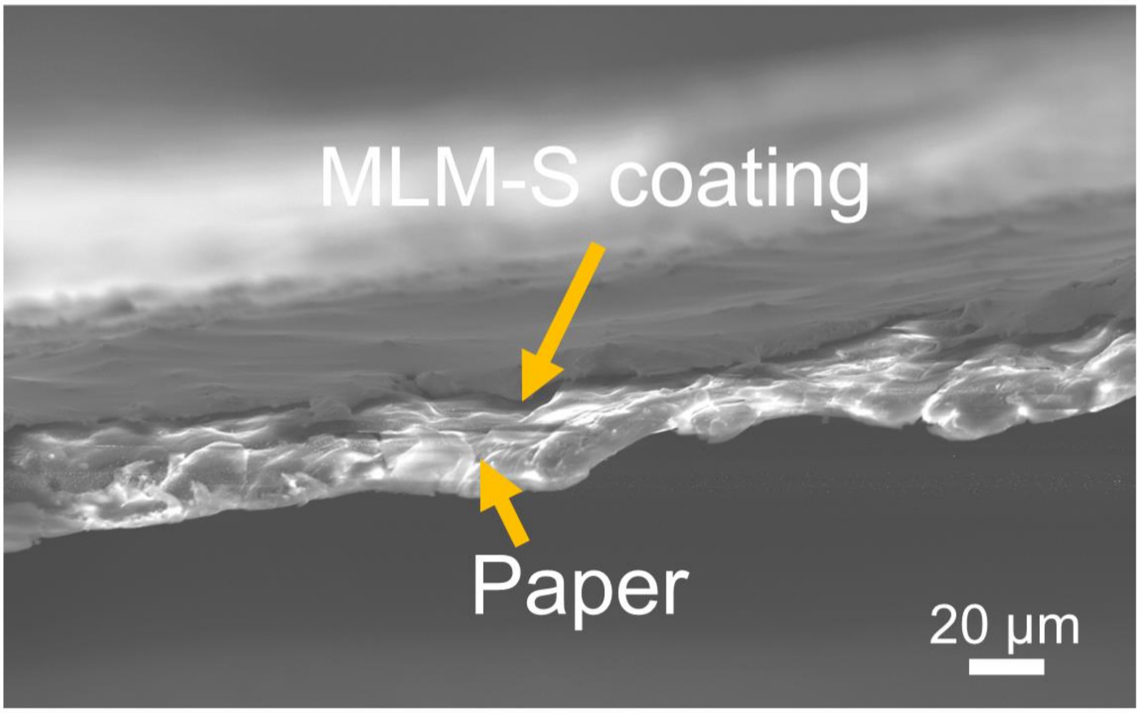

Supplementary Fig. 15 | Microscopic characterization of the MXene/liquid metal composite prepared by solvent-assisted dispersion (MLM-S). SEM image of the cross-section of MLM-S coating applied to a piece of paper.

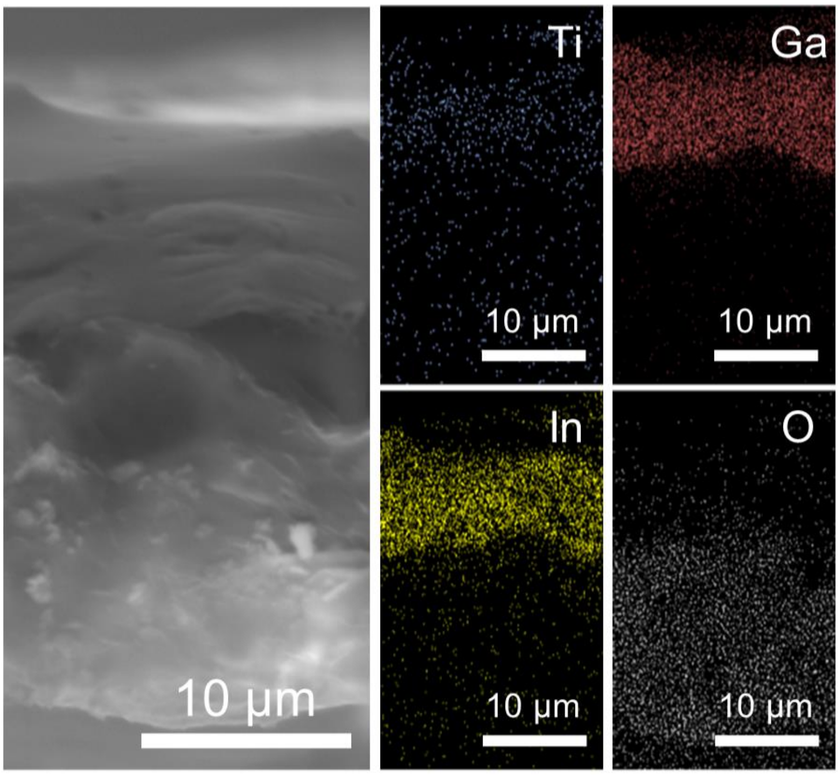

Supplementary Fig. 16 | Microscopic characterization of the MXene/liquid metal composite prepared by solvent-assisted dispersion (MLM-S). SEM image and EDS mapping of the cross-section of the MLM-S coating on the paper.

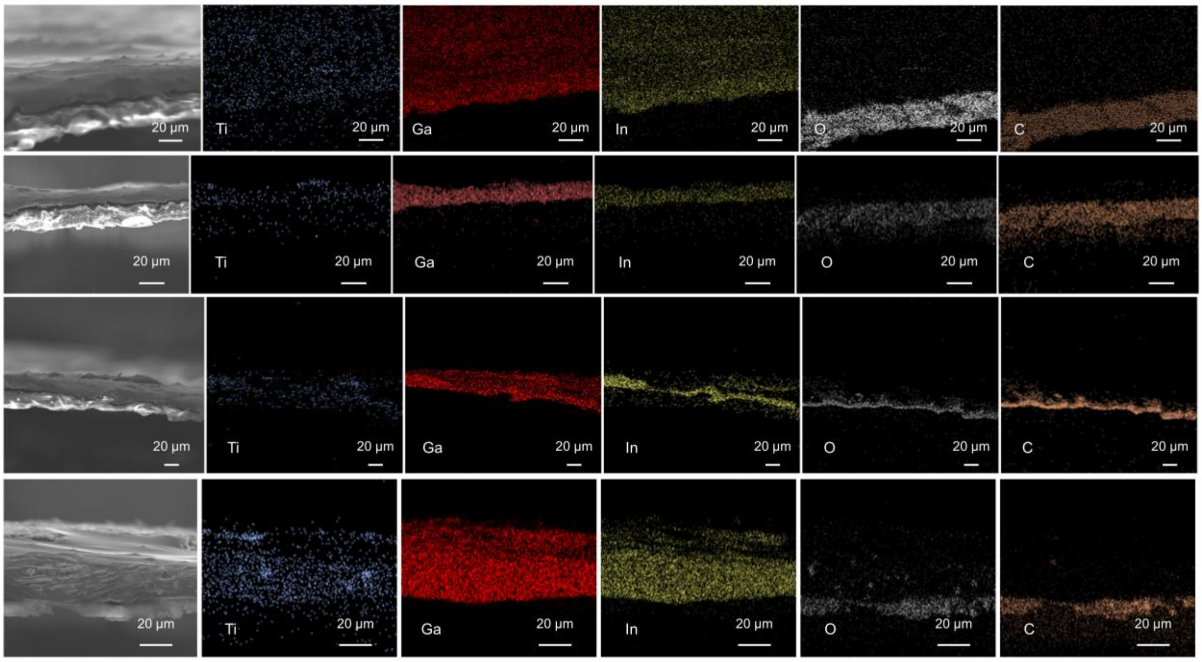

Supplementary Fig. 17 | Microscopic characterization of the MXene/liquid metal composites prepared by solvent-assisted dispersion (MLM-S). SEM and EDS images of cross sections with different thicknesses of MLM-S coatings.

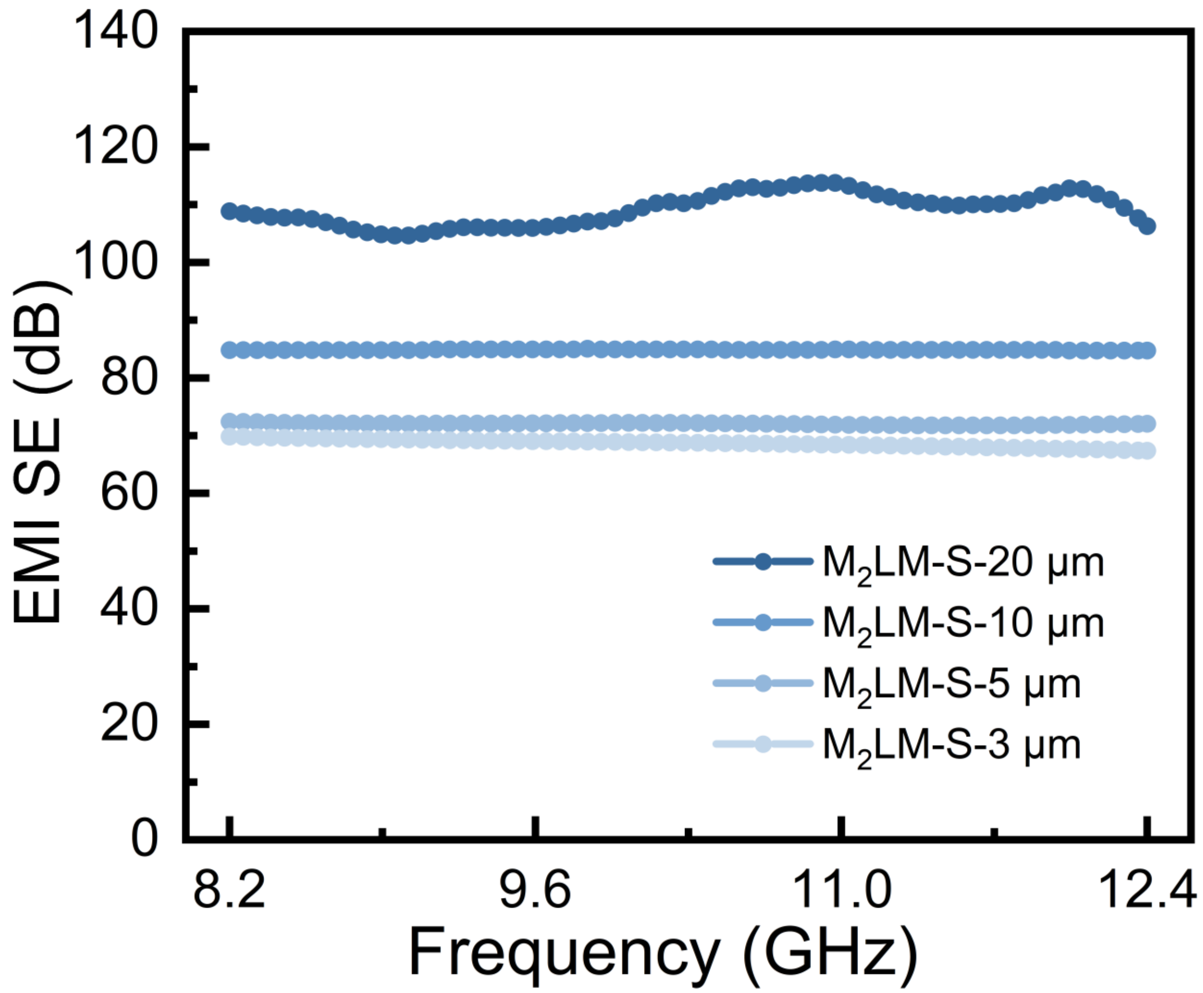

Supplementary Fig. 18 | Electromagnetic shielding performance of the MXene/liquid metal composite prepared by solvent-assisted dispersion (MLM-S). EMI SE of M_2LM-S at different thicknesses.

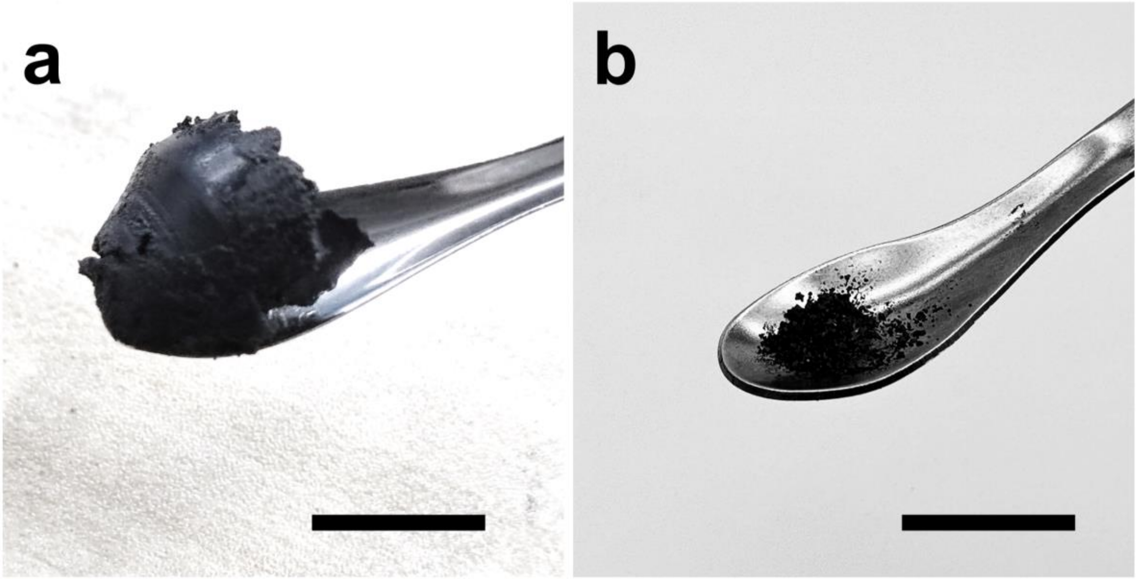

Supplementary Fig. 19 | Comparison between MXene paste and powder. Optical photographs of 100 mg **(a)** MXene paste and **(b)** powder. Scale bar, 10 mm.

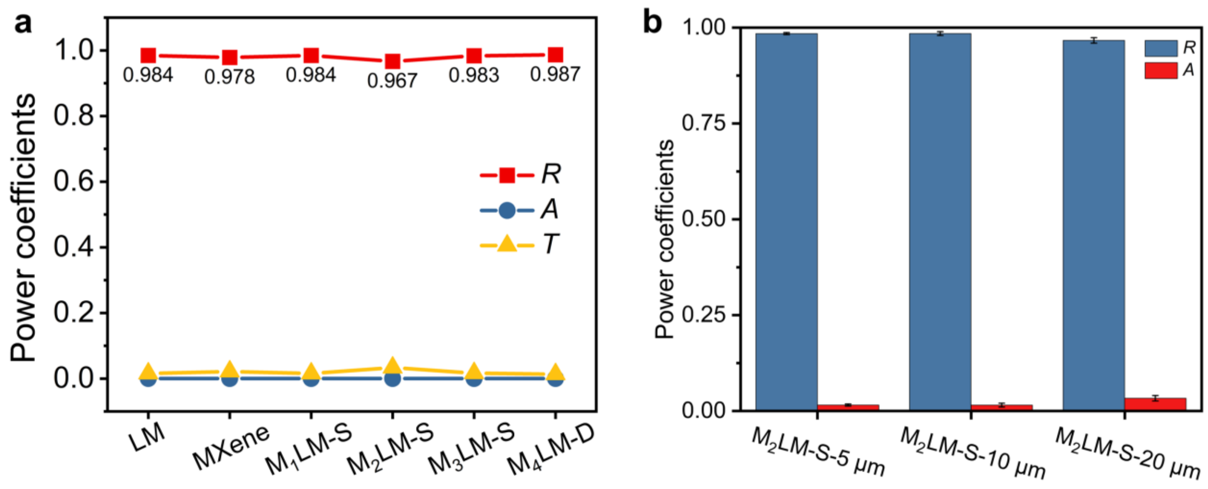

Supplementary Fig. 20 | The power coefficients of the MXene/liquid metal composite prepared by solvent-assisted dispersion (MLM-S). (a) *R*-*A*-*T* coefficients of LM, MXene, M_1LM-S, M_2LM-S, M_3LM-S, and M_4LM-D at 20 μm. **(b)** R-A-T coefficients of M_2LM-S at different thicknesses. Error bars are calculated from the mean ± standard deviation (SD) of 201 scanning points in the X-band for each sample.

Apparently, among the 20 μm thick MLM-S coatings, M_2LM-S has the largest *A* of 0.033 and the smallest *R* of 0.967, demonstrating that the enhancement of absorption by the biphasic conducting network formed by MXene doping can provide a significant improvement in the EMWs attenuation of the coating. Moreover, the *A*-value increases while the *R*-value decreases as the thickness of the coating increases in Supplementary Fig. 20b, demonstrating the enhanced absorption attenuation of EMWs by the internal MXene network.

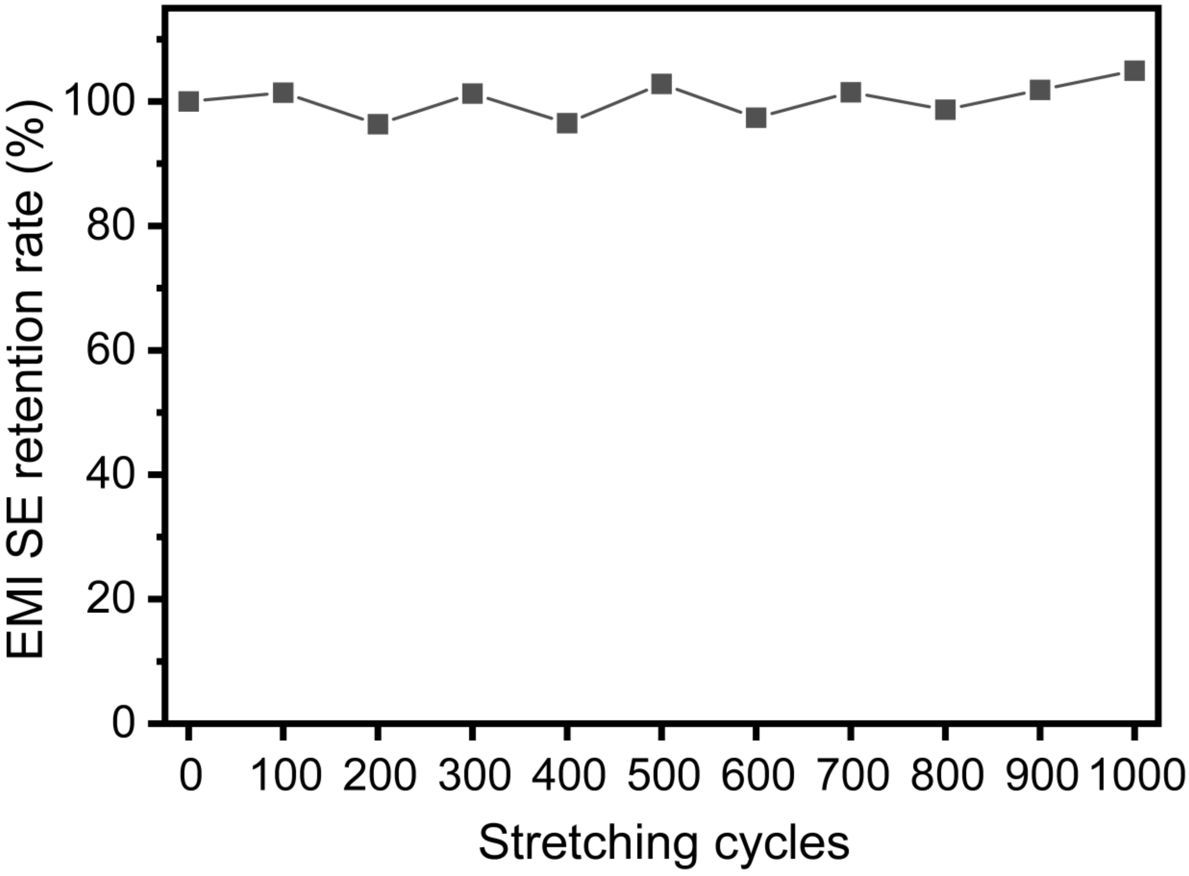

Supplementary Fig. 21 | Cyclic tensile test of the coating of the MXene/liquid metal composite prepared by solvent-assisted dispersion (MLM-S). EMI SE of Ecoflex films coated with M_2LM-S after stretching 1000 cycles.

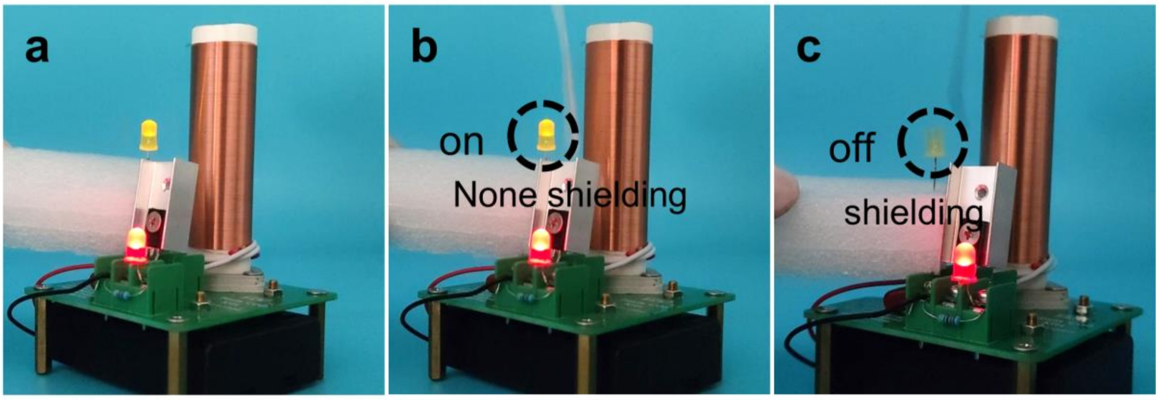

Supplementary Fig. 22 | Tesla coil shielding experiment. (a) Optical images of a Tesla coil. Optical images of EMI shielding of a Tesla coil with **(b)** a piece of paper and **(c)** MLM-S coating paper.

The bulb remained bright when the uncoated paper was inserted between the bulb and the induction coil. In contrast, when the MLM-coated paper was inserted, the bulb went out, indicating that the coating disrupted the resonance conditions of the device, suggesting that MLM could be practically applied[10,11].

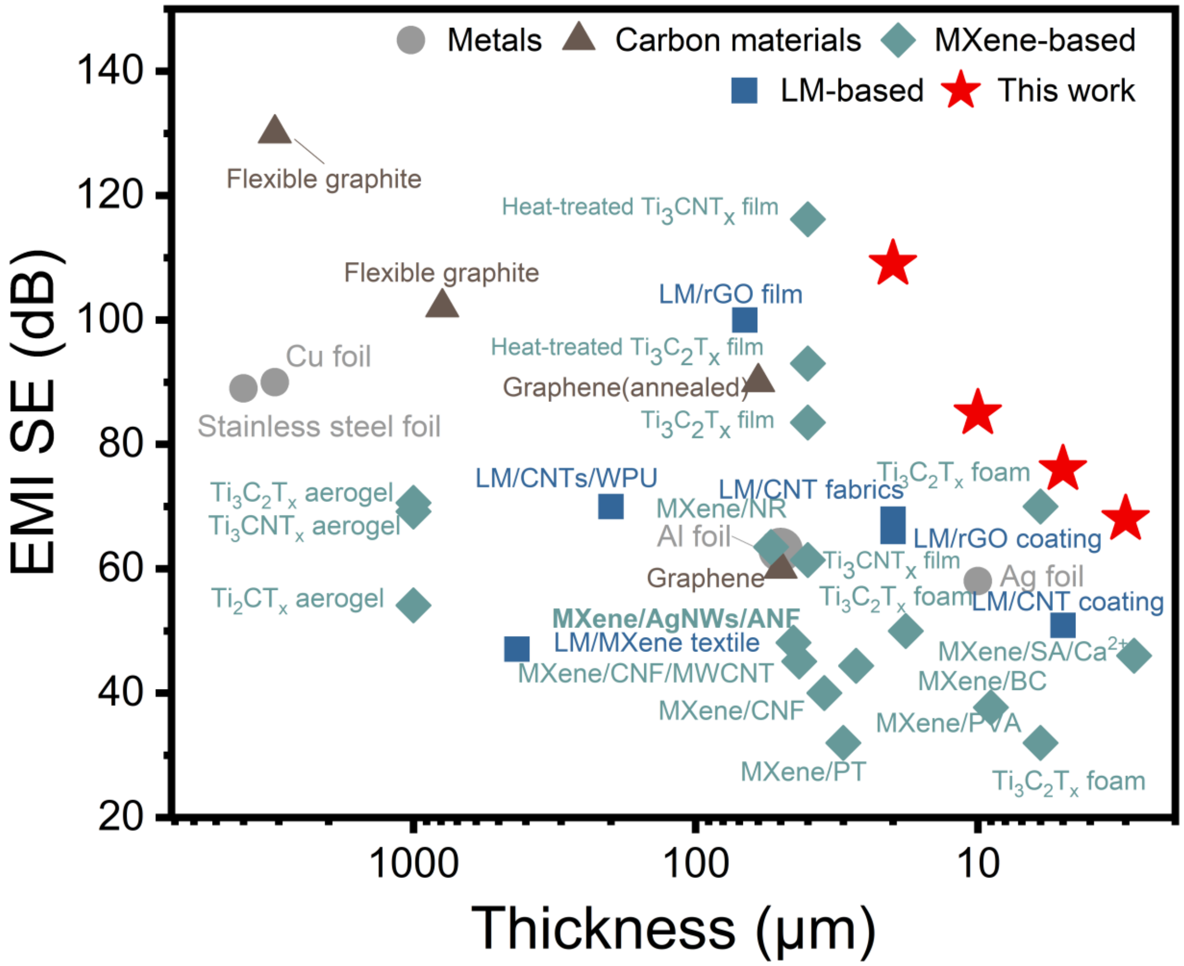

Supplementary Fig. 23 | Comparison of electromagnetic shielding properties between literature and samples in this work. Comparison of EMI SE between M_2LM-S coating and other EMI shielding materials published in the literature.

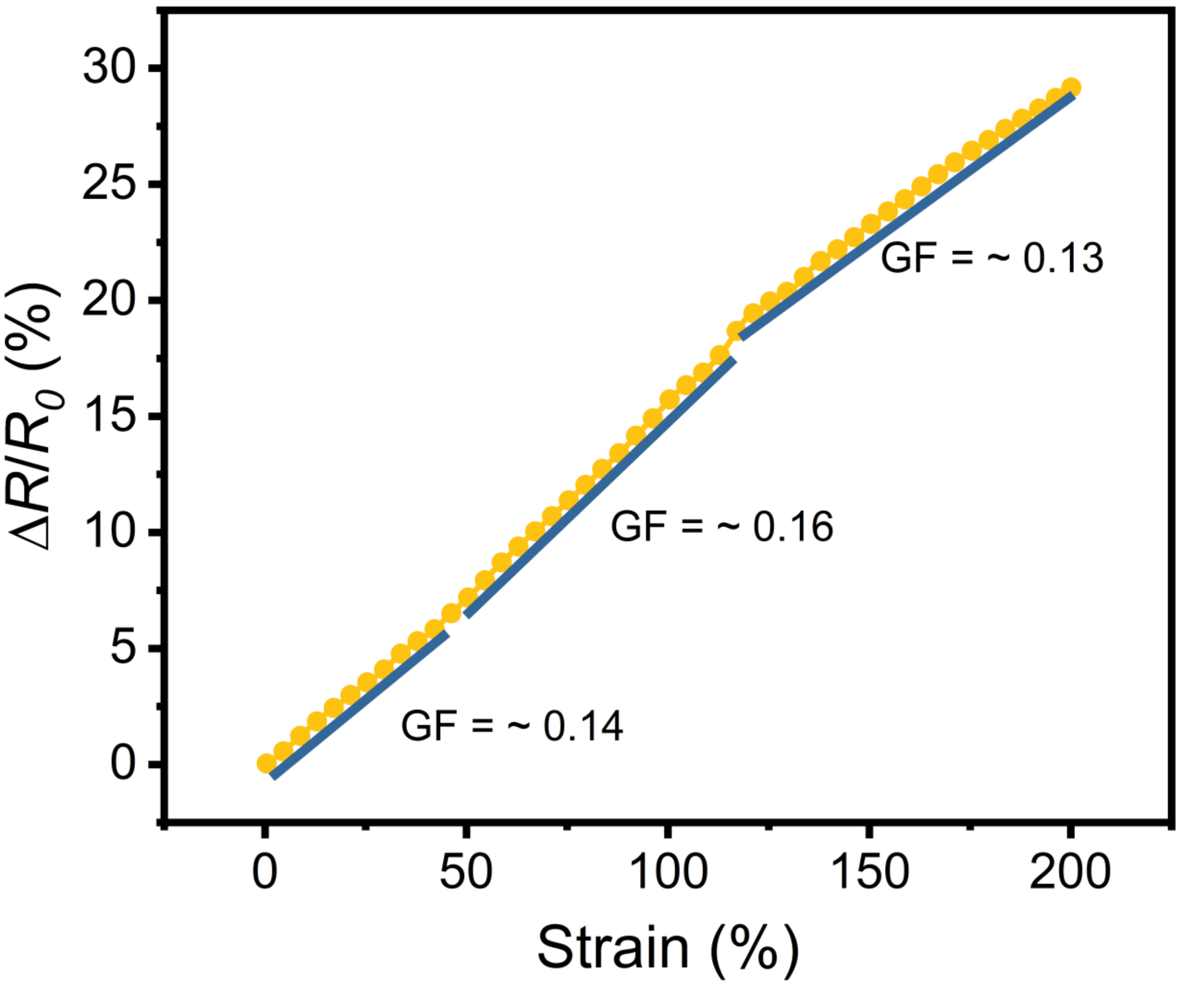

Supplementary Fig. 24 | GF factor of the sample. Variation of the relative resistance of M_2LM-S coating on Ecoflex surface with tensile strain.

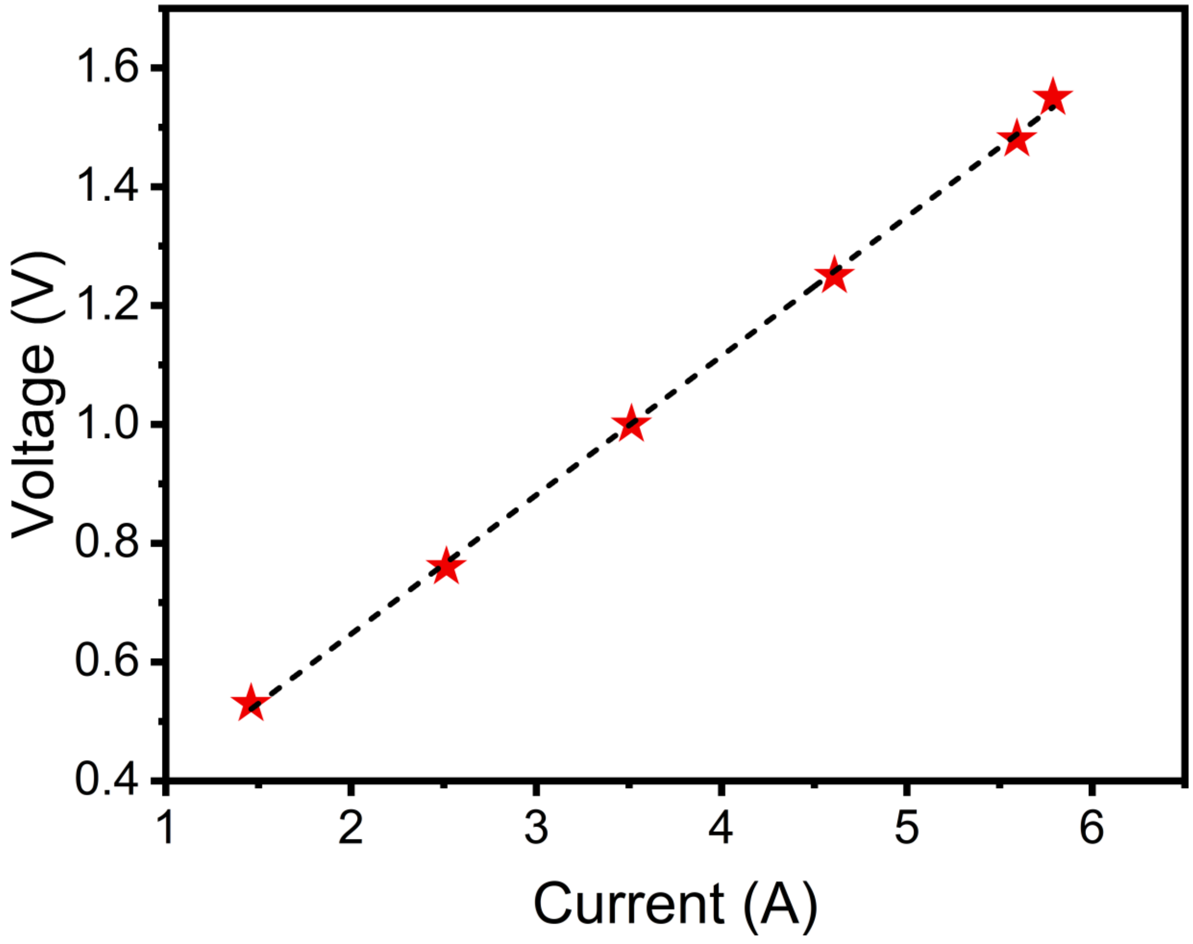

Supplementary Fig. 25 | The voltammetry characteristic curve of the sample. *I*−*V* curve of MLM-S heater at ascending driving voltages.

The nearly linear *I*−*V* curve of the MLM-S coating indicates a stable coating resistance, following Ohm's Law.

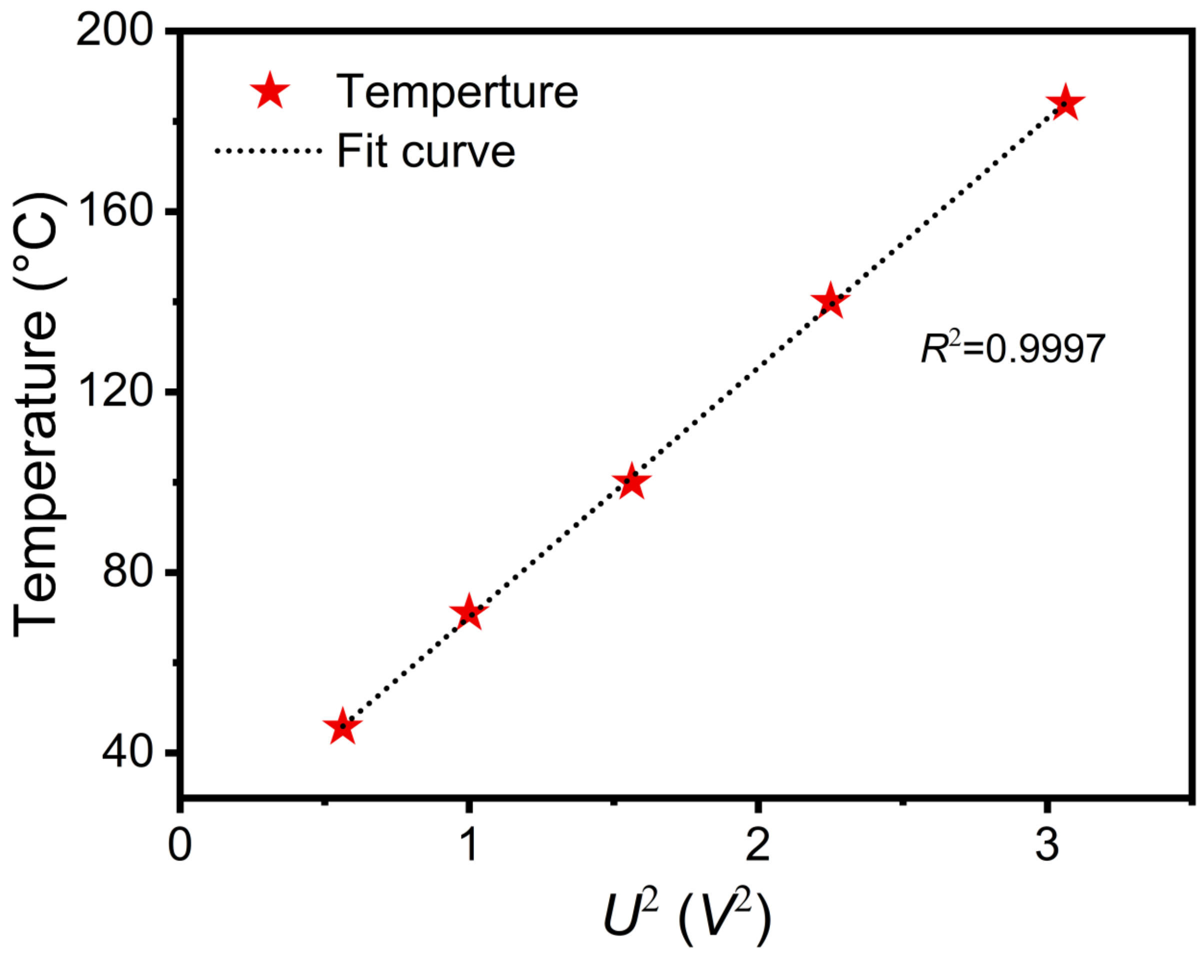

Supplementary Fig. 26 | The variation curve of U^2 of the sample with temperature. Temperature of the MLM-S coating under different operation voltages.

The high compliance of Joule's Law ($Q = U^2R^{-1}t$) with the linear relationship between temperature and the square of input voltage demonstrates its high heating controllability by varying the input voltage.

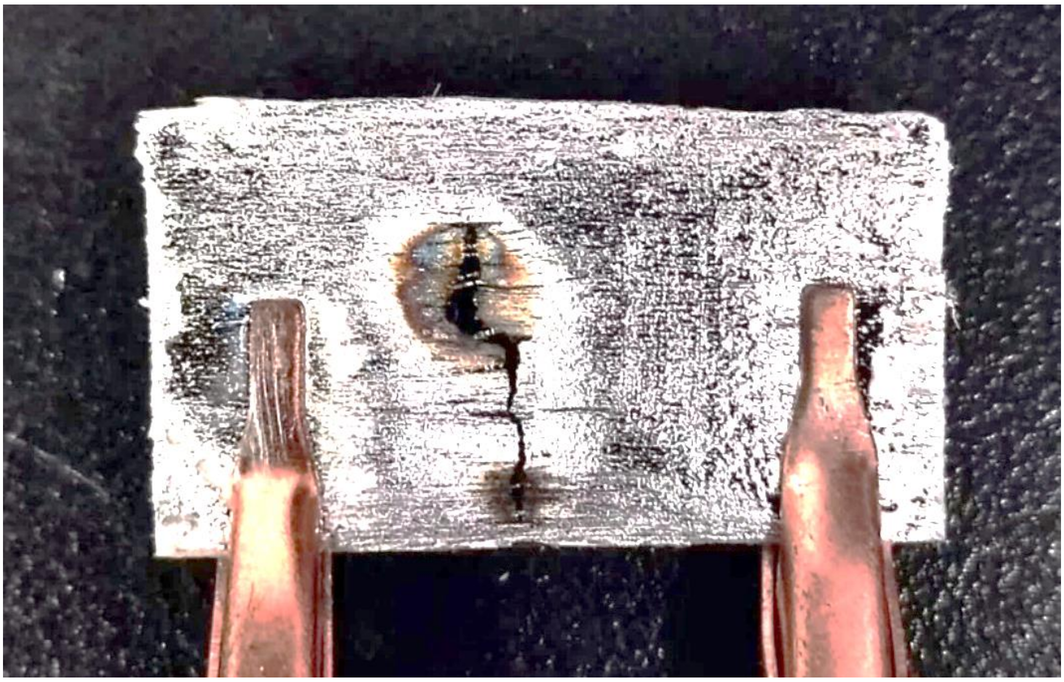

Supplementary Fig. 27 | A board burned through by excessive voltage. The wood substrate burns through at a voltage of 2.5 V.

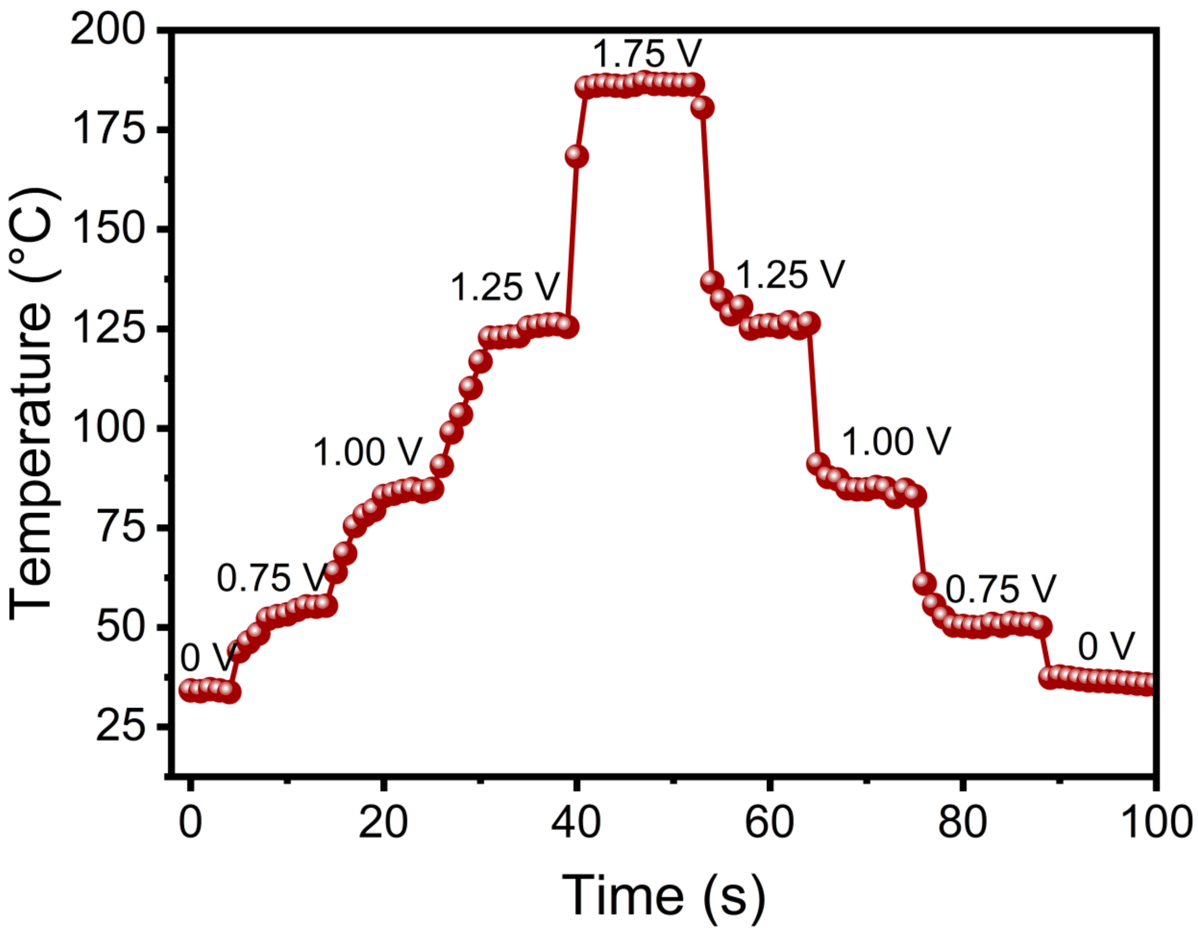

Supplementary Fig. 28 | A cycle of voltage from 0 V to 1.75 V and then back to 0 V. Saturation temperature evolution curve during voltage adjustment.

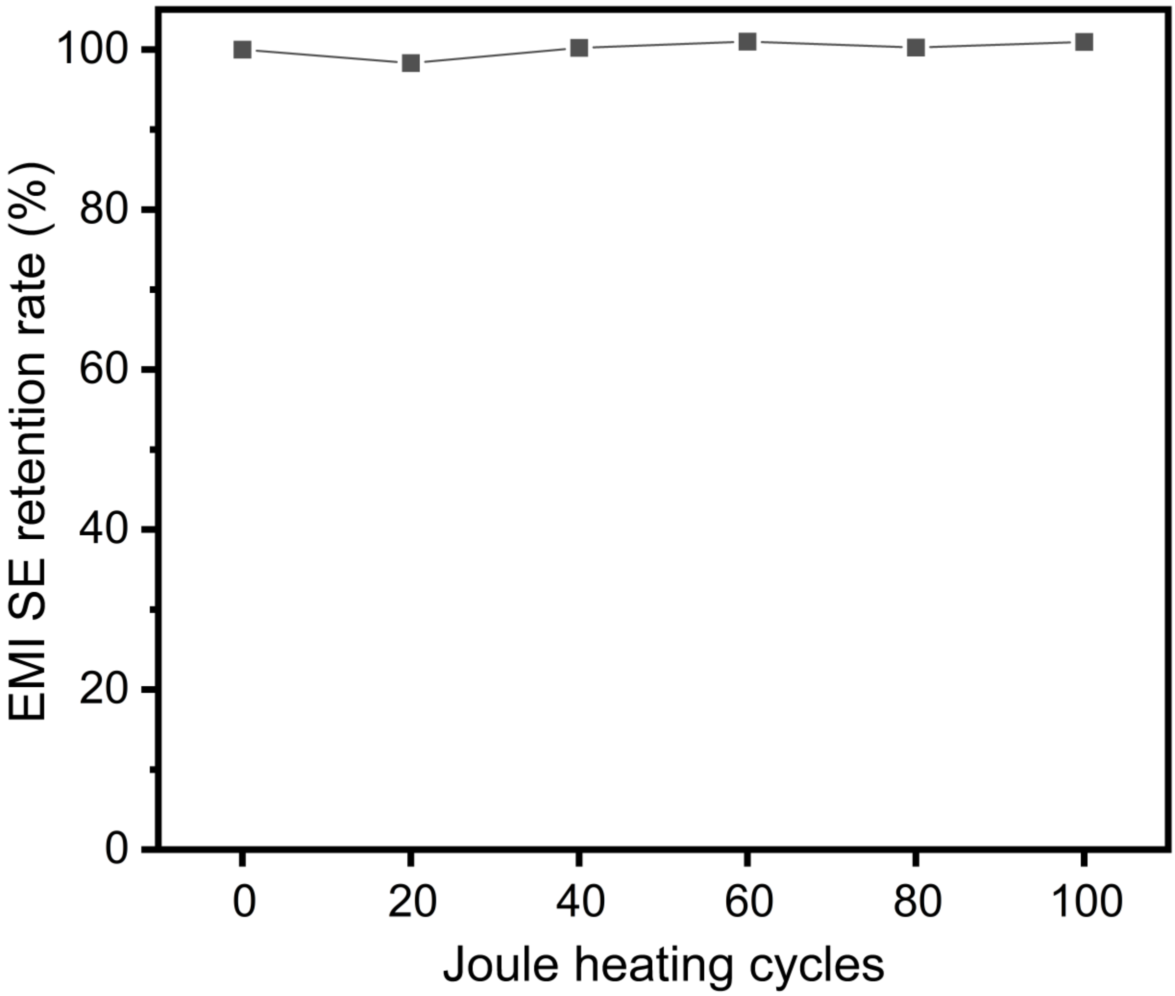

Supplementary Fig. 29 | Stability of electromagnetic shield after Joule heating cycle. EMI SE of the M_2LM-S coating after 100 joule heating cycle.

Supplementary Table 1 SAD was used to inject the conventional unmixed fillers into LM

Filler	Mass loading	Result
Multi-walled carbon nanotubes	1.0 wt%	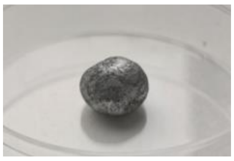
Graphene	1.0 wt%	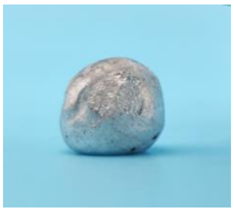
Nono Fe_3O_4	2.0 wt%	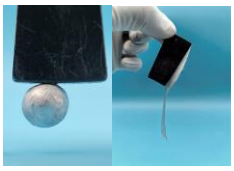

Supplementary Table 2 Comparison of EMI shielding performance for various materials.

Materials	Thickness (μm)	EMI SE in X-band [dB]	SSE_t [dB cm^2 g^{-1}]	Ref.
Cu foil	3100	90	32	12
Stainless steel foil	4000	89	28	12
Ag foil	10	58	5576	13
Al foil	50	63	4630	13
Graphene	50	60	6000	14
Graphene(annealed)	60	90	7500	15
Flexible graphite	790	102	645.6	16
	3100	130	209.7	16
MXene/CNF	35	40	7029	17
MXene/AgNWs/ANF	45	48.1	10688.9	18
MXene/BC	9	37.7	21429	19
MXene/NR	54	63.5	18989.8	20
MXene/CNF/MWCNT	43	45.1	19543.17	21
MXene/PVA	27	44.4	9343	22
MXene/PT	30	32.0	4085.92	23
$Ti_3C_2T_x$ film	40	83.5	9488.64	24
Heat-treated $Ti_3C_2T_x$ film	40	93.0	10568.2	24
Ti_3CNT_x film	40	61.4	6977.3	24
Heat-treated Ti_3CNT_x film	40	116.2	13204.6	24
$Ti_3C_2T_x$ aerogel	1000	70.6	64182	25
Ti_3CNT_x aerogel	1000	69.2	62909	25

Ti_2CT_x aerogel	1000	54.1	49182	25
$Ti_3C_2T_x$ foam	6	32	136752	26
$Ti_3C_2T_x$ foam	18	50	69444	26
$Ti_3C_2T_x$ foam	6	70	53030	26
Mxene/SA/Ca^{2+}	2.8	46	58929	27
LM/CNT fabrics	20	68	8500	28
LM/CNT coating	5	50.9	22622.2	29
LM/rGO coating	20	66	24812	6
LM/rGO film	67	100	7462.7	30
LM/MXene textile	430	47.1	1095.3	31
LM/CNTs/WPU	200	70	5984.4	32
LM/MXene plasticine	3	68	59336.8	This work
	5	76	39790.6	
	10	85	22251.3	
	20	109	14267.1	

Supplementary Table 3 The mass of LM and MXene in MLM composites and their EMI shielding performance in X-band.

Samples	LM mass (g)	MXene powder mass(g)	Silver nanosheets mass(g)	MXene paste mass(g)	EMI SE (dB) -20 μm
LM	/	/	/	/	~59.6
MXene film	/	/	/	/	~65.4
M_4LM-D	4.00	0.16	/	/	~66.0
M_6LM-D	4.00	0.24	/	/	~64.7
M_8LM-D	4.00	0.32	/	/	~63.7
M_{10}LM-D	4.00	0.40	/	/	~63.8
M_{20}LM-D	4.00	0.80	/	/	/
Ag_{10}LM-D	4.00	/	0.40	/	~40.5
M_1LM-S	4.00	/	/	0.04	~89.7
M_2LM-S	4.00	/	/	0.08	~109.2
M_3LM-S	4.00	/	/	0.12	~89.2

References

1 Kresse, G. & Furthmüller, J. Efficient iterative schemes for ab initio total-energy calculations using a plane-wave basis set. *Phys. Rev. B* **54**, 11169-11186, doi:10.1103/PhysRevB.54.11169 (1996).

2 Perdew, J. P., Burke, K. & Ernzerhof, M. Generalized Gradient Approximation Made Simple. *Physical Review Letters* **77**, 3865-3868, doi:10.1103/PhysRevLett.77.3865 (1996).

3 Momma, K. & Izumi, F. VESTA 3 for three-dimensional visualization of crystal, volumetric and morphology data. *Journal of Applied Crystallography* **44**, 1272-1276 (2011).

4 Stukowski, A. Visualization and analysis of atomistic simulation data with OVITO–the Open Visualization Tool. *Modelling and Simulation in Materials Science and Engineering* **18**, 015012, doi:10.1088/0965-0393/18/1/015012 (2010).

5 Wang, J., Ma, X., Zhou, J., Du, F. & Teng, C. Bioinspired, High-Strength, and Flexible MXene/Aramid Fiber for Electromagnetic Interference Shielding Papers with Joule Heating Performance. *ACS Nano*, doi:10.1021/acsnano.2c01323 (2022).

6 Wang, C. *et al.* A general approach to composites containing nonmetallic fillers and liquid gallium. *Science Advances* **7**, eabe3767, doi:doi:10.1126/sciadv.abe3767 (2021).

7 Rueda, M. M. *et al.* Rheology and applications of highly filled polymers: A review of current understanding. *Progress in Polymer Science* **66**, 22-53, doi:https://doi.org/10.1016/j.progpolymsci.2016.12.007 (2017).

8 Kim, H. *et al.* Shape-Deformable and Locomotive MXene (Ti3C2Tx)-Encapsulated Magnetic Liquid Metal for 3D-Motion-Adaptive Synapses. *Advanced Functional Materials* **33**, doi:10.1002/adfm.202210385 (2022).

9 Cademartiri, L. *et al.* Electrical Resistance of AgTS–S(CH2)n−1CH3//Ga2O3/EGaIn Tunneling Junctions. *The Journal of Physical Chemistry C* **116**, 10848-10860, doi:10.1021/jp212501s (2012).

10 Wang, H. *et al.* Multifunctional Filler-Free PEDOT:PSS Hydrogels with Ultrahigh Electrical Conductivity Induced by Lewis-Acid-Promoted Ion Exchange. *Adv Mater*, e2302919, doi:10.1002/adma.202302919 (2023).

11 Guo, H. *et al.* Electrospun TaC/Fe3C–Fe carbon composite fabrics for high efficiency of electromagnetic interference shielding. *Composites Communications* **31**, 101130, doi:https://doi.org/10.1016/j.coco.2022.101130 (2022).

12 Shui, X. & Chung, D. D. L. Nickel filament polymer-matrix composites with low surface impedance and high electromagnetic interference shielding effectiveness. *Journal of Electronic Materials* **26**, 928-934, doi:10.1007/s11664-997-0276-4 (1997).

13 Ji, H. *et al.* Lightweight and flexible electrospun polymer nanofiber/metal nanoparticle hybrid membrane for high-performance electromagnetic interference shielding. *NPG Asia Materials* **10**, 749-760, doi:10.1038/s41427-018-0070-1 (2018).

14 Zhang, L. *et al.* Preparation and characterization of graphene paper for electromagnetic interference shielding. *Carbon* **82**, 353-359, doi:https://doi.org/10.1016/j.carbon.2014.10.080 (2015).

15 Xi, J. *et al.* Graphene aerogel films with expansion enhancement effect of high-performance electromagnetic interference shielding. *Carbon* **135**, 44-51, doi:https://doi.org/10.1016/j.carbon.2018.04.041 (2018).

16 Luo, X. & Chung, D. D. L. Electromagnetic interference shielding reaching 130 dB using flexible graphite. *Carbon* **34**, 1293-1294, doi:https://doi.org/10.1016/0008-6223(96)82798-9 (1996).

17 Zhou, B. *et al.* Flexible, Robust, and Multifunctional Electromagnetic Interference Shielding Film with Alternating Cellulose Nanofiber and MXene Layers. *ACS Appl. Mater. Interfaces* **12**, 4895-4905, doi:10.1021/acsami.9b19768 (2020).

18 Zeng, Z., Li, W., Wu, N., Zhao, S. & Lu, X. Polymer-Assisted Fabrication of Silver Nanowire Cellular Monoliths: Toward Hydrophobic and Ultraflexible High-Performance Electromagnetic Interference Shielding Materials. *ACS Appl. Mater. Interfaces* **12**, 38584-38592, doi:10.1021/acsami.0c10492 (2020).

19 Wan, Y. *et al.* Ultrathin, Strong, and Highly Flexible Ti3C2Tx MXene/Bacterial Cellulose Composite Films for High-Performance Electromagnetic Interference Shielding. *ACS Nano* **15**, 8439-8449, doi:10.1021/acsnano.0c10666 (2021).

20 Wang, Y., Liu, R., Zhang, J., Miao, M. & Feng, X. Vulcanization of Ti3C2Tx MXene/natural rubber composite films for enhanced electromagnetic interference shielding. *Applied Surface Science* **546**, 149143, doi:https://doi.org/10.1016/j.apsusc.2021.149143 (2021).

21 Qian, K. *et al.* Yarn-ball-shaped CNF/MWCNT microspheres intercalating Ti3C2Tx MXene for electromagnetic interference shielding films. *Carbohydr. Polym.* **254**, 117325, doi:https://doi.org/10.1016/j.carbpol.2020.117325 (2021).

22 Jin, X. *et al.* Flame-retardant poly(vinyl alcohol)/MXene multilayered films with outstanding electromagnetic interference shielding and thermal conductive

performances. *Chemical Engineering Journal* **380**, 122475, doi:https://doi.org/10.1016/j.cej.2019.122475 (2020).

23 Wang, Y. *et al.* MXene-coated conductive composite film with ultrathin, flexible, self-cleaning for high-performance electromagnetic interference shielding. *Chemical Engineering Journal* **412**, 128681, doi:https://doi.org/10.1016/j.cej.2021.128681 (2021).

24 Iqbal, A. *et al.* Anomalous absorption of electromagnetic waves by 2D transition metal carbonitride Ti3CNT x (MXene). *Science* **369**, 446-450, doi:10.1126/science.aba7977 (2020).

25 Han, M. *et al.* Anisotropic MXene Aerogels with a Mechanically Tunable Ratio of Electromagnetic Wave Reflection to Absorption. *Advanced Optical Materials* **7**, 1900267, doi:https://doi.org/10.1002/adom.201900267 (2019).

26 Liu, J. *et al.* Hydrophobic, Flexible, and Lightweight MXene Foams for High-Performance Electromagnetic-Interference Shielding. *Adv Mater* **29**, doi:10.1002/adma.201702367 (2017).

27 Wan, S. *et al.* Strong sequentially bridged MXene sheets. *Proc Natl Acad Sci U S A* **117**, 27154-27161, doi:10.1073/pnas.2009432117 (2020).

28 Wang, J. *et al.* Liquid metal/CNT nanocomposite coated cotton fabrics for electromagnetic interference shielding and thermal management. *Cellulose* **29**, 8907-8918, doi:10.1007/s10570-022-04821-1 (2022).

29 Yi, S.-Q. *et al.* CNT-assisted design of stable liquid metal droplets for flexible multifunctional composites. *Composites Part B: Engineering* **239**, doi:10.1016/j.compositesb.2022.109961 (2022).

30 Sun, Y. *et al.* Slippery Graphene-Bridging Liquid Metal Layered Heterostructure Nanocomposite for Stable High-Performance Electromagnetic Interference Shielding. *ACS Nano*, doi:10.1021/acsnano.3c02975 (2023).

31 Yi, P. *et al.* MXene-Reinforced Liquid Metal/Polymer Fibers via Interface Engineering for Wearable Multifunctional Textiles. *ACS Nano* **16**, 14490-14502, doi:10.1021/acsnano.2c04863 (2022).

32 Nguyen, V.-T., Nguyen, Q.-D., Min, B. K., Yi, Y. & Choi, C.-G. Ti3C2Tx MXene/carbon nanotubes/waterborne polyurethane based composite ink for electromagnetic interference shielding and sheet heater applications. *Chemical Engineering Journal* **430**, doi:10.1016/j.cej.2021.133171 (2022).
