# Supplementary material for: Malleable, printable, bondable, and highly conductive MXene/liquid metal plasticine with improved wettability
Source: Nat Commun. 2024 Jul 20;15:6138. doi: 10.1038/s41467-024-50541-4 (PMC11271265; doi:10.1038/s41467-024-50541-4)
Supplement: Supplementary file 3 — Description of Additional Supplementary Files [file 41467_2024_50541_MOESM3_ESM.pdf]

Description of Additional Supplementary Files

File Name: Supplementary Movie 1

Description: 1 X-ray microscopy scanning and 3D reconstruction.

File Name: Supplementary Movie 2

Description: 3D reconstruction of MXene (blue) and LM (orange).

File Name: Supplementary Movie 3

Description: The malleable and bondable MLM-S Supplementary Movie 4 MLM-S in high viscosity condition and can hold swabs.

File Name: Supplementary Movie 5

Description: Printing of MLM-S by a flexible electronic printer Supplementary Movie 6 MLM-S coated on PDMS for in-situ stretching and twisting.

File Name: Supplementary Movie 7

Description: Applying MLM-S onto a paper by scraping.

File Name: Supplementary Movie 8

Description: Joule heating properties of MLM-S coatings under cyclic stretching and bending.
